# Green synthesis of gold nanoparticles utilizing *Raphanus sativus* root extract: characterization and in vitro investigation of its antioxidant, antimicrobial and anticancer impact

**DOI:** 10.1038/s41598-026-60345-9

**Published:** 2026-07-03

**Authors:** Yara Mohamed Taha, Mohamed Ali El-Desouky, Heba Ali Abd El-Rahman, Maha Hanafy Mahmoud

**Affiliations:** 1 Biochemistry Lab, Faculty of Science, Cairo University, Cairo, 12613 Egypt; 2 Chemistry department, Biochemistry division, Faculty of Science, Cairo University, Cairo, 12613 Egypt; 3 Assistant professor of Embryology, Zoology Department, Faculty of Science, Cairo University, Cairo, 12613 Egypt; 4 Professor of Biochemistry and Nutrition, Department of Nutrition and Food Science, Institute of Food Industries and Nutrition Research, National Research Centre, Cairo, 12622 Egypt

**Keywords:** Gold nanoparticles, Red radish root, Green synthesis, Antioxidant, Antimicrobial, Anticancer, Biochemistry, Biological techniques, Biotechnology, Cancer, Chemistry, Drug discovery, Microbiology, Nanoscience and technology, Plant sciences

## Abstract

Gold nanoparticles (AuNPs) are recently investigated as substantial tools in cancer therapy due to their biocompatibility and tunable surface properties. *Raphanus sativus* or red radish root (RRR), a Cruciferaceae vegetable, has notable medicinal and nutritional value owing to its highly polyphenolic content and antioxidant property. The aim of this study is to synthesize the gold nanoparticles by a green method using the *Raphanus sativus* root extract and to study its antioxidant, antimicrobial and anticancer impact. The resultant RRR-AuNPs were characterized by TEM, UV/Vis spectroscopy, DLS, Zeta potential, XRD, AFM, BET and pore size analysis and assessed for antioxidant, antimicrobial and anticancer activity. The greenly synthesized gold nanoparticles showed ruby red color with a distinct absorption peak at 526 nm. The resultant AuNPs were spherical with an average size of 31 nm and average zeta potential of -36.8 mV. Results clearly demonstrated that the RRR-AuNPs possess high total polyphenolic content and potent antioxidant activity (DPPH). Moreover, RRR-AuNPs exhibited a considerable antibacterial efficacy against *Staphylococcus aureus* (80.9% inhibition) and *Escherichia coli* (78.22% inhibition) with a uniform MIC value of 0.977 µg/mL. Furthermore, RRR-AuNPs reduced the viability of Caco-2 and HepG2 cancer cells to 31.9% and 26.4%, respectively, at 250 µg/mL. Notably, a remarkable Antimicrobial Selectivity Index (SI) of 303.04 was established, confirming a toxicity over 300-fold higher toward bacterial pathogens than toward normal HEK-293 cells. These findings highlight RRR-AuNPs as a promising, biocompatible platform with potent antioxidant, antimicrobial, and anticancer properties for biomedical applications.

**Supplementary Information:**

The online version contains supplementary material available at 10.1038/s41598-026-60345-9.

## Introduction

Metallic nanoparticles represent one of the most versatile categories of nanomaterials owing to their widespread implementation in electronics, chemistry, pharmaceutics, and medical sectors. Among these various metal nanoparticles, gold nanoparticles (AuNPs) which offer a wide range of prospective applications due to their distinct qualities, that differ a lot from their bulk form^1^. Consequently, AuNPs have been extensively integrated into nanomedicine for diagnostic, imaging, therapeutic, drug carrier and delivery systems because of their biocompatibility, customizable surface chemistry, and unique optical and physicochemical properties^2^.

AuNPs are synthesized using a variety of techniques, including chemical, biological, and physical processes. Traditional physical and chemical synthesis strategies are frequently expensive, demand extreme operating conditions and require hazardous stabilizing chemicals that restrict their applications in human diagnosis and clinical medicine^3,4^.

To mitigate these limitations, green synthesis methodologies have attracted intense research focus over recent decades. These green routes represent simple, eco-friendly, low-cost and sustainable alternatives that utilize non-toxic solvents, such as water, to be suitable for human wellness and environment^5^. This phytogenic synthesis strategy uses natural bioactive compounds derived from microorganisms (e.g., bacteria, fungi, algae) or plant extracts to serve as reducing, capping, and stabilizing agents that convert gold ions (Au^+ 1^) to gold metal (Au^0^)^2,6^.

Prior literature confirms that specific functional groups within these natural extracts, particularly hydroxyl groups, provide the reducing potential required for nanomaterial nucleation. Furthermore, these co-extracted phytochemicals preserve nanoparticle stability and enhance its biological activity. Biogenic AuNPs display diverse biomedical functionalities, including anti-inflammatory, antimicrobial, anticancer, and photothermal activities, alongside specialized roles in cosmetics, biosensing, and drug delivery^7^.

Recent investigations have documented the successful green synthesis of AuNPs using diverse botanical sources, including extracts from *Aloe vera*, *Punica granatum*, *Acacia nilotica* leaves, green coconut shells, *Nerium oleander*, and *Abroma augusta*, among others^8^.

A highly promising yet distinct plant candidate is the radish (*Raphanus sativus* L.), a globally cultivated and consumed root vegetable belonging to the Brassicaceae family. Because of its high content of fibre, moisture, and vitamins A and C, radishes offer significant nutritional and therapeutic value^9^. It exhibits well-documented pharmacological benefits, including digestion-promoting, anti-inflammatory, antimicrobial, anticancer, antioxidant, and hemostatic activities. These diverse biological effects are mediated by an abundance of phytochemicals, such as isothiocyanates, glucosinolates, anthocyanins, flavonoids, saponins, alkaloids, and phenolic acids^9^.

However, the utilization of *Raphanus sativus* L. root (red radish root; RRR) as a green capping and reducing agent for AuNP synthesis remains largely unexplored. Current literature reveals a notable scarcity of research leveraging RRR extracts for metallic nanoparticle fabrication compared to alternative plant parts. To bridge this literature gap, this study comprehensively investigates the synthesis, structural optimization, and morphological characterization of RRR-derived AuNPs. Furthermore, the clinical relevance of these biosynthesized nanoparticles is demonstrated through the evaluation of their antioxidant profiles, alongside their targeted antibacterial and anticancer activities.

## Materials and methods

### Preparation of RRR extract

Fresh roots of *Raphanus sativus* (red radish) were purchased from a local market in Cairo, Egypt. The plant material was authenticated at the Herbarium of Cairo University. The red radish root was washed many times by distilled water, dried in oven at 50 °C and was milled using a mechanical grinder (Braun, Germany) to obtain fine powder. Ten grams of powdered dried root was boiled in 300 mL distilled water for 15 min to form extract. The Extract is filtered by mash then by Whatman filter paper No.1 and after this step; the prepared extract of 44.4 gm/l is ready to be used for the synthesis of Au-NPs^10^.

### HPLC (high performance liquid chromatography) for the water extract of red radish root

HPLC analysis was carried out using an Agilent 1260 series. The separation was carried out using Zorbax Eclipse Plus C8 column (4.6 mm x 250 mm i.d., 5 μm). The mobile phase consisted of water (A) and 0.05% trifluoroacetic acid in acetonitrile (B) at a flow rate 0.9 ml/min. The mobile phase was programmed consecutively in a linear gradient as follows: 0 min (82% A); 0–1 min (82% A); 1–11 min (75% A); 11–18 min (60% A); 18–22 min (82% A); 22–24 min (82% A). The multi-wavelength detector was monitored at 280 nm. The injection volume was 5 µl of the sample. The column temperature was maintained at 40 °C^11^.

### Synthesis of RRR-AuNPs

Green synthesis of AuNPs was carried out according to Zhao et al., (2021)^12^ with some modifications. A volume of 50 mL of RRR extract was mixed with 1 mM HAuCl4 solution. PH was adjusted to 10–11 using sodium hydroxide (NaOH) to maintain the optimum pH that stabilizes the gold nanoparticles and prevents agglomeration. Then, the mixture was heated to 60–80 °C to allow the formation of RRR-AuNPs to proceed for a designated period, usually 1–3 h. The final concentration of the prepared AuNPs was 1 mg/mL.

### DPPH radical scavenging activity

The effect of the extract on free radical of 1,1 diphenyl-2-picrylhydrazyl (DPPH) was assessed according to the method of Alvares and Furtado, (2021)^13^ with some modifications. Briefly, a 0.5 mL aliquot of either the RRR water extract, RRR-AuNPs, or ascorbic acid solution which served as the positive control (50 mg/L, obtained from El-Nasr Company for Intermediate Chemicals), was thoroughly mixed with 1 mL of a methanolic DPPH solution (0.2 mM). The mixture was incubated in the dark for 30 min at room temperature. The absorbance of the tested samples was measured at 517 nm by a spectrophotometer (Shimadzu, UV-2401 PC, Australia). For the control, the test was done in the same manner, but sample solution is replaced by ethanol. DPPH scavenging capacity percent of the tested samples was measured as a decrease in the absorbance and was calculated by using the following equation:

$$Scavenging{\text{ }}activity{\text{ }}\left( \% \right) = \frac{{Ac - As}}{{Ac}} \times 100$$

Where Ac and As are the absorbance of the control and the sample, respectively.

### Evaluation of total phenolic content

The total phenolic compounds (TPC) were determined for boiled water extract of RRR and RRR-AuNPs solutions by the Folin-Ciocalteu test according to Mahmoud et al., (2022)^14^. A volume of 250 µL of each solution was mixed with 3.5 mL of distilled water then 250µL of Folin-Ciocalteu reagent was added. Next, 250 µL of 20% sodium carbonate solution was added. All samples, tested in triplicates, were mixed using vortex and incubated at 40 °C in a water-bath for 20 min. The absorbance of the resultant blue color was read against the blank standard at 765 nm wavelength. The TPC was calculated regarding to gallic acid concentration. Results were expressed as mg of gallic acid per dried plant weight. Blank was prepared by using 250 µL of 80% ethanol instead of RRR extract or RRR-AuNPs.

### Characterization of gold nanoparticles

#### UV/Visible spectroscopy analysis

UV/Visible spectrometer was used to characterize the synthesized AuNPs for their absorption peak; the absorbance spectra were obtained using UV/Vis spectroscopy at a wavelength range 200 to 800 nm. (Shimadzu, Japan)^15^.

#### Transmission electron microscope (TEM)

Morphological characteristics, including distribution, size, and shape, were analyzed by using high resolution-TEM (HRTEM, JEOL TEM-2100, Japan) with 20 X magnification and 250 kV accelerating voltage. Primarily, RRR-AuNPs has been prepared by sonicated infusion using sonication propunder with one pulse/second pulse rate at 85% amplitude power maximum temperature for 30 min. Finally, 50 micron was subjected to TEM grade for 5 h in air dry^16^. The selected area electron diffraction (SAED) analysis was carried out to examine the crystalline nature of RRR-AuNPs.

#### Fourier transform infrared spectroscopy analysis (FT-IR)

The functional groups of the RRR water extract and the biomolecular capping agents of the synthesized RRR-AuNPs were characterized via FT-IR spectroscopy (JASCO, Tokyo, Japan) across a wavenumber range of 4000–400 cm^− 1^.

#### Energy dispersive X-ray spectroscopy (EDX)

The elemental composition of RRR-AuNPs was determined using Energy Dispersive X-ray (EDX) spectroscopy (EDAX APEX, AMETEK, USA).

#### Atomic force microscope (AFM)

AFM- (SPM AA 3000, USA) was used to describe qualitative and quantitative information on numerous physical characteristics such as morphology, size, surface texture and roughness Providing 3D visualization capability^17^.

#### Dynamic light scattering (DLS)

DLS is a spectroscopy method that is used to determine the size distribution of particles in solution or suspension. DLS was conducted by particle size analyzer that was manufactured by Malvern Panalytical Ltd. Model of NanoSight NS500 (United Kingdom)^18^.

#### Zeta potential determination

Zeta potential is used to describe the dispersion stability. Zeta potential of the nanoparticles was measured by zeta sizer analyzer that was manufactured by Malvern Panalytical Ltd. Model of NanoSight NS500(United Kingdom)^19^.

#### X-ray diffraction (XRD)

XRD analysis was performed to identify the crystalline structure of the synthesized gold nanoparticles according to Ismail et al., (2021)^20^. The XRD pattern of the RRR-AuNPs sample was recorded by an X-beam diffractometer (XRD, D8-Find, Bruker, Madison, WI, USA) equipped with CuKα radiation (λ = 1.5418 Å) radiation filter, working at a current of 40 MA, voltage of 40 kV and step filter 0.01º.

#### Surface area analysis [brunauer- emmett-teller (BET) and pore size screening]

Surface area and pore size were determined according to Hassan et al., (2022)^21^ using surface area and pore size analyzer (Quanta chrome model of NOVA touch 2LX, USA).

### Antimicrobial activity of RRR and RRR-AuNPs test (percent of inhibition)

Antimicrobial activity of water extract of RRR and RRR-AuNPs is detected by percent of inhibition test according to Xiang et al., (2018)^22^. *Escherichia coli* (*E. coli*) and *Staphylococcus aureus* (*S. aureus*) were utilized to assess the percent of inhibition of bacterial growth of the RRR water extract and greenly synthesized gold nanoparticles (RRR-AuNPs) using agar-count plating method. All the strains were obtained from the Microbial Resources Centre, Faculty of Agriculture, Ain Shams University, Egypt. A bacterial suspension of McFarland 0.5 standard solutions of *S. aureus* and *E. coli* were prepared in Mueller-Hinton broth medium and incubated for 16–24 h at 37 °C. In the next day, 200 µL of each prepared bacterial suspensions were added into 4 sterile tubes each of which contained 200 µL of Mueller-Hinton broth and 100 µL of RRR extract or RRR-AuNPs or Amoxicillin (5 mg/ml positive control) or DMSO (negative control). Then the tubes incubated at 37 °C for 24 h. In the third day, 10-fold serial dilution of the incubated suspensions was done and 20 µL of diluted bacterial solution of each sample was cultured on the surface of solidified nutrient agar plates. Finally, the agar plates were incubated at 37 °C for 24 h. The bacterial colonies (CFU) on the plates were observed and counted by naked eyes. The antibacterial efficacy was calculated as follows:

$$Percent{\text{ }}of{\text{ }}Growth{\text{ }}Inhibition{\text{ }}(\% ){\text{ }} = \frac{{(Number{\text{ }}of{\text{ }}CFUs{\text{ }}in{\text{ }}control{\text{ }}group{\text{ }} - {\text{ }}Number{\text{ }}of{\text{ }}CFUs{\text{ }}in{\text{ }}\exp erimental{\text{ }}group) \times 100}}{{Number{\text{ }}of{\text{ }}CFUs{\text{ }}in{\text{ }}control{\text{ }}group}}$$

### Determination of minimum inhibitory concentration (MIC) and minimum bactericidal concentration (MBC)

Two to five isolated colonies of *S. aureus* and *E. coli* were selected from the fresh agar plate and were transferred into tubes containing 3–4 ml of sterile Mueller-Hinton broth (MHB) medium. The resulting bacterial suspension was homogenized well and incubated at 35–37 °C for 3 h. The turbidity of each suspension was adjusted to be equivalent to McFarland Standard 0.5 at 600 nm. After that, RRR water extract (10 mg/mL), RRR-AuNPs (1 mg/mL), and amoxicillin (1 mg/mL; positive control) were two-fold serially diluted using sterile MHB medium. The standardized bacterial suspensions were then subjected to a ten-fold serial dilution and inoculated in fixed, equal volumes into each tube containing the diluted RRR extract, RRR-AuNPs or amoxicillin. The tubes were incubated at 37 °C for 16–20 h. The MIC is defined as the lowest concentration of the antimicrobial agent that inhibits visible growth of the tested isolate as observed with the unaided eye^23^. The Minimum Bactericidal Concentration (MBC) was determined by subculturing aliquots from broth tubes that exhibited no visible growth onto fresh, treatment-free nutrient agar plates and incubated at 37 °C for 16–24 h. MBC was identified as the lowest concentration of the agent that achieved a 99.9% reduction of the initial bacterial inoculum^24^.

### Time killing assay

Time-killing assay was carried out to evaluate the time and concentration-dependent antimicrobial profiles of the RRR water extract, RRR-AuNPs and amoxicillin against *S. aureus* and *E. coli*. A bacterial suspension was adjusted to 0.5 McFarland standard and diluted 1:100 in sterile broth to obtain an initial concentration of approximately 1 × 10^6^ CFU/mL. Subsequently, equal volumes of bacterial suspension and respective treatments prepared at four times their minimum inhibitory concentration (4 MIC) were mixed to each other. These treatments included the RRR water extract, RRR-AuNPs, amoxicillin antibiotic (positive control), and a negative control consisting of sterile deionized water. This equal-volume mixing resulted in a final treatment concentration of (2 MIC). The mixtures were then incubated at 37 °C for designated time intervals: 0 min, 30 min, 1 h, 2 h, 4 h, 6 h, and 24 h. At each specific incubation time, 10 µL of the treatment/bacteria mixture was ten-fold serially diluted using sterile phosphate buffer solution (PBS). After that, 20 µL of the diluted suspension was inoculated on Muller-Hinton agar (MHA) in duplicate and incubated for 18–21 h at 37 °C for bacterial counting. The results of the total viable bacterial counts were expressed as log10 CFU/mL^25^.

### Cell viability assay (MTT test)

The cell Viability assay was performed at The Regional Centre For Mycology And Biotechnology, AL Azhar University, Cairo, Egypt. Briefly, Caco-2 (human colon carcinoma), HepG2 (human hepatocellular carcinoma) and HEK-293 (normal human embryonic kidney) cells line were plated in 96-well plates at a concentration of 1 × 10^5^ cells/mL and incubated at 37 °C in a humidified 5% CO2/95% air atmosphere for 24 h to enable a complete monolayer sheet of cells to develop. Growth medium was decanted and attached cells were washed twice with washing media. The attached monolayer cells were treated with 100 µL of two-fold diluted RRR extract (100 mg/mL) or RRR-AuNPs solutions (1000 µg/mL) prepared in RPMI medium with 2% serum (maintenance medium) except the 3 wells of control which contained maintenance medium only. Doxorubicin was used as a positive control (10 mg/mL) and prepared in the same serial dilutions under the same experimental conditions. The plate was then incubated at 37 °C and checked.

After that, 20 µL of 5 mg/mL MTT solution (Biobasic Canada INC) was added to each well and mixed using a shaker at 150 rpm for 5 min. The plate was further incubated at 37 °C and 5% CO2 for 4 h to allow the MTT to be metabolized. After incubation, the media was decanted, and the plate was dried on towels to remove residue. Formazan (MTT metabolic product) was resuspended in 200 µL DMSO and mixed using on a shaker at 150 rpm for 5 min to ensure that formazan was mixed into its solvent. The optical density was read at 560 nm and the background was subtracted at 620 nm. Optical density should be directly correlated with cell quantity. Half-maximal inhibitory concentration (IC_50_) was presented as the mean ± SD. All experiments conducted at least in triplicate^26–28^.

### Antimicrobial selectivity index (SI)

The Selectivity Index (SI) was calculated to evaluate the therapeutic safety margin of the RRR-AuNPs and RRR water extract^29^. The SI represents the ratio of the toxic concentration to the effective bioactive concentration and was determined using the following formula:

$$SI = \frac{{IC_{{50}} (Normal \;cells)}}{{MIC(Pathogenic\;bacteria)}}$$

### Statistical analysis

Statistical analysis was performed using the SPSS software version “22” for Windows. Data are depicted as mean ± standard deviation (SD). Comparisons between two independent groups were conducted using an independent Sample T-test. A *p* value < 0.05 was considered statistically significant. Comparisons between more than two groups were analysed using One-Way analysis of variance (ANOVA) followed by the Duncan multiple comparisons test. *P* < 0.05 was considered statistically significant, *p* < 0.01 was considered highly significant and *p* < 0.000 was considered very highly significant.

## Results

### HPLC of the water extract of red radish root

**Fig. 1:**
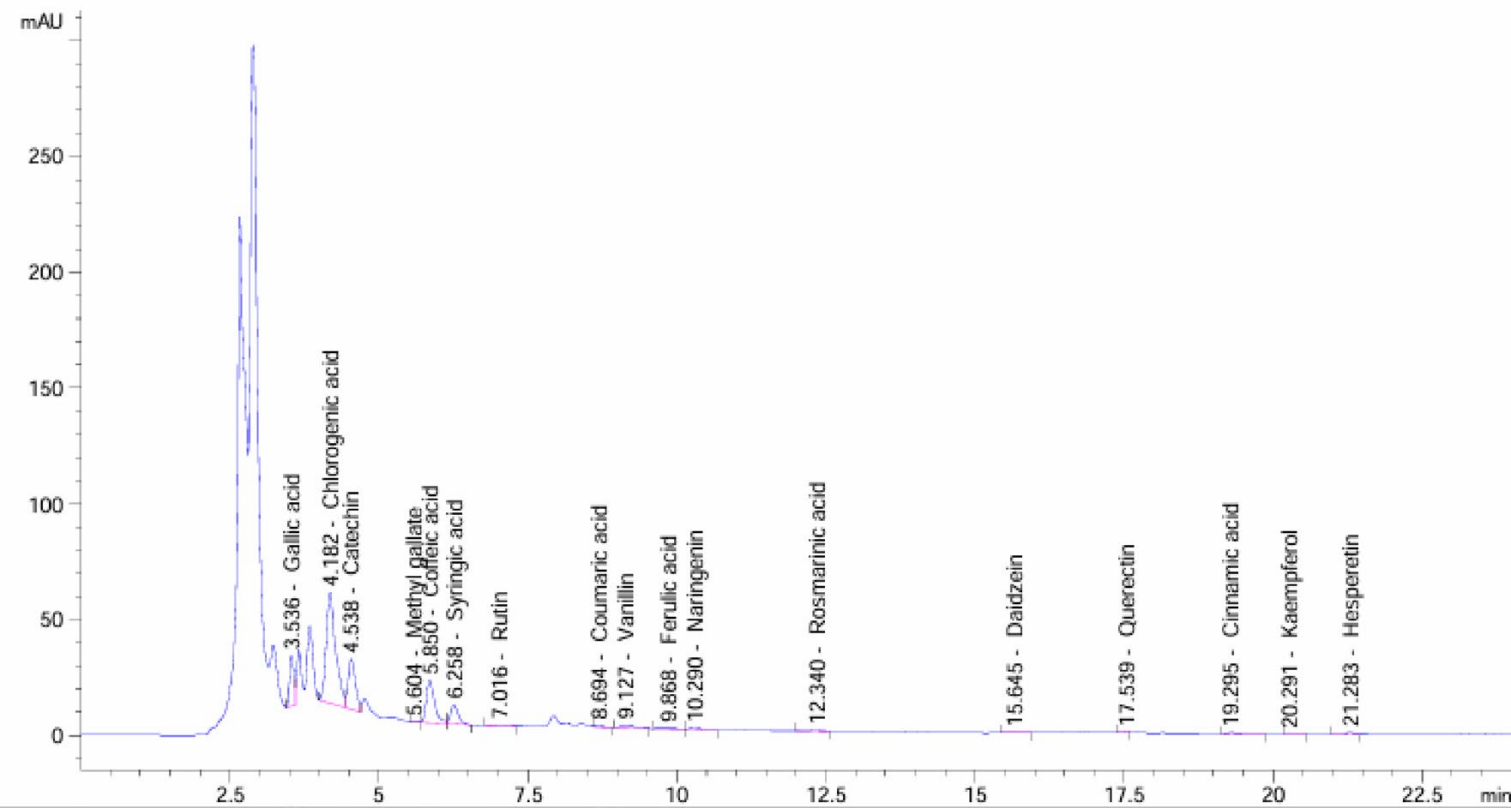
HPLC profile of phenolic compounds of water extract of red radish root at 280 nm.

Analysis by HPLC to water extract of red radish root revealed the presence of different phenolic compounds. Using 19 standard phenolic compounds to be detected in RRR extract (Fig. 11), a high amount of catechins, gallic acid and caffeic acid were found in RRR water extract. In addition, a valuable amount of syringic acid, rutin, naringenin, vanillin, rosmarinic acid, ferulic acid, hesperetin, quercetin, coumaric acid, cinnamic acid, kaempferol, daidzein, chlorogenic acid and methyl gallate were detected in the water extract of RRR (Table 1) (Fig. 1).

**Table 1:** Differential pattern of distinct phenolic compounds identified in water extract of red radish root as detected by HPLC.

Phenolic compounds	Concentration (µg/1gm dry wt)
Gallic acid	143.6
Chlorogenic acid	1.04
Catechin	627
Methyl gallate	0.75
Caffeic acid	209.9
Syringic acid	88.8
Pyro catechol	0
Rutin	16.65
Ellagic acid	0
Coumaric acid	3
Vanillin	10.05
Ferulic acid	6.45
Naringenin	15.3
Rosmarinic acid	9.45
Daidzein	1.05
Quercetin	3.6
Cinnamic acid	1.65
Kaempferol	1.5
Hesperetin	4.5

### DPPH radical scavenging activity

**Fig. 2:**
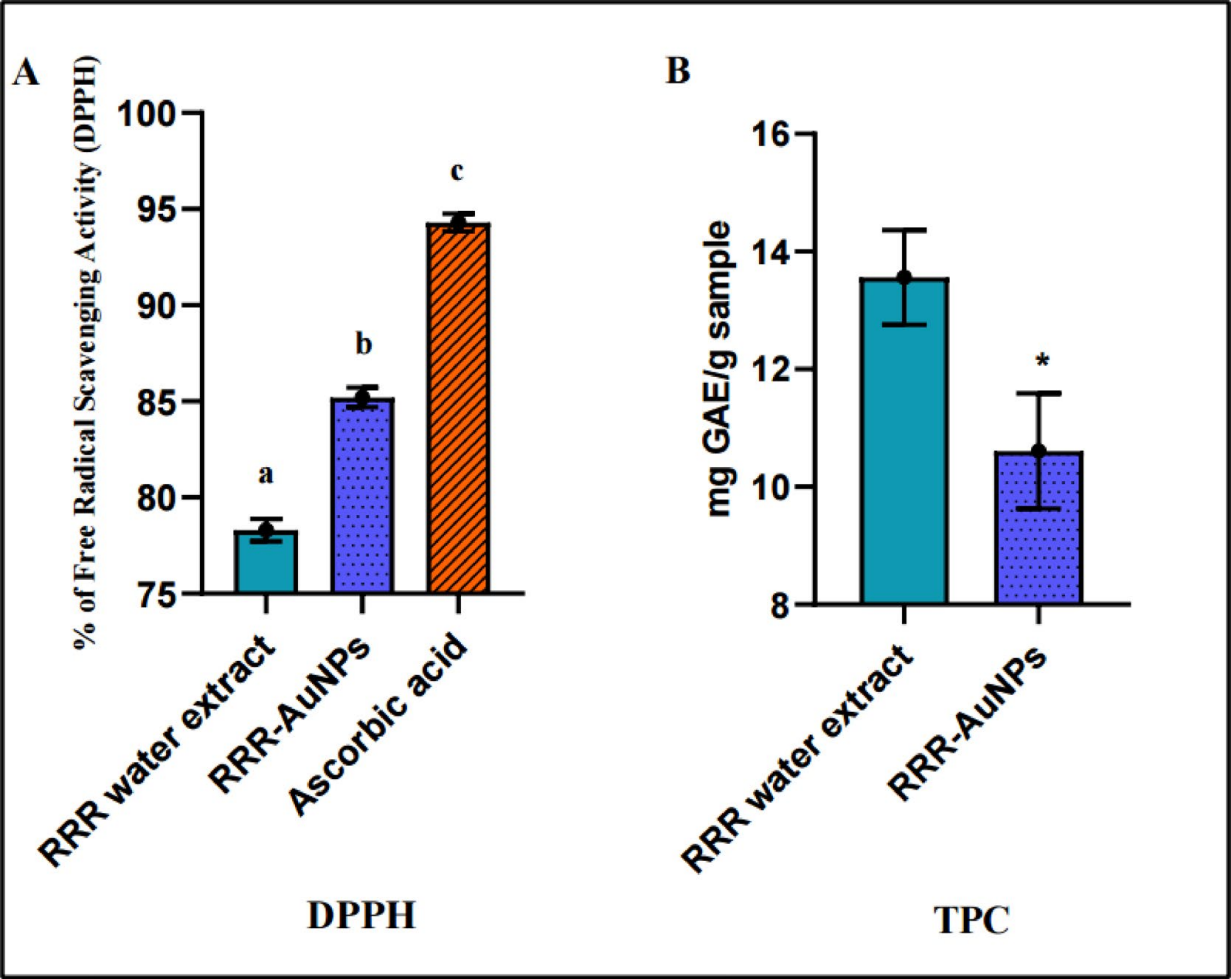
Antioxidant activity assessed by DPPH assay and total phenolic content of both RRR water extract and RRR−AuNPs **(A)** Percentage of free radical scavenging activity (DPPH); Statistical significance was measured by ANOVA test. Data are represented as Mean±SD. Bars that share the same letters within one series are not significant, while bars that share different letters within one series are significant and the level of significance was considered at *p* < 0.05. **(B)** TPC values expressed as mg gallic acid equivalents per g sample. Data are represented as Mean±SD. Statistical significance was determined using the unpaired Student’s t−test, with results considered significant at *p* < 0.05 (*).

The free radical scavenging capacity of RRR extract and RRR-AuNPs was determined by using a stable, synthetic free radical molecule of 1,1-diphenyl-2-picrylhydrazyl (DPPH). Water extract of RRR and RRR-AuNPs showed high free radical scavenging capacity percent, 78.3% and 85.2%, respectively, confirming their antioxidant activity (*P* = 0.000). These values were significantly lower than the 94.3% recorded for the ascorbic acid positive control (Fig. 2A).

### Evaluation of total phenolic content

Total phenolic content of RRR water extract and RRR-AuNPs were identified through the Folin-Ciocalteu assay. The results of TPC were expressed as mg GAE per one gram of dried plant weight and evaluated using the gallic acid standard curve. The analysis revealed a high concentration of phenols for both water extract and RRR- AuNPs which were 13.56 and 10.61 mg GAE/1 gm dried plant weight, respectively (*P* = 0.016) (Fig. 2B).

### UV/Visible spectroscopy analysis

**Fig. 3:**
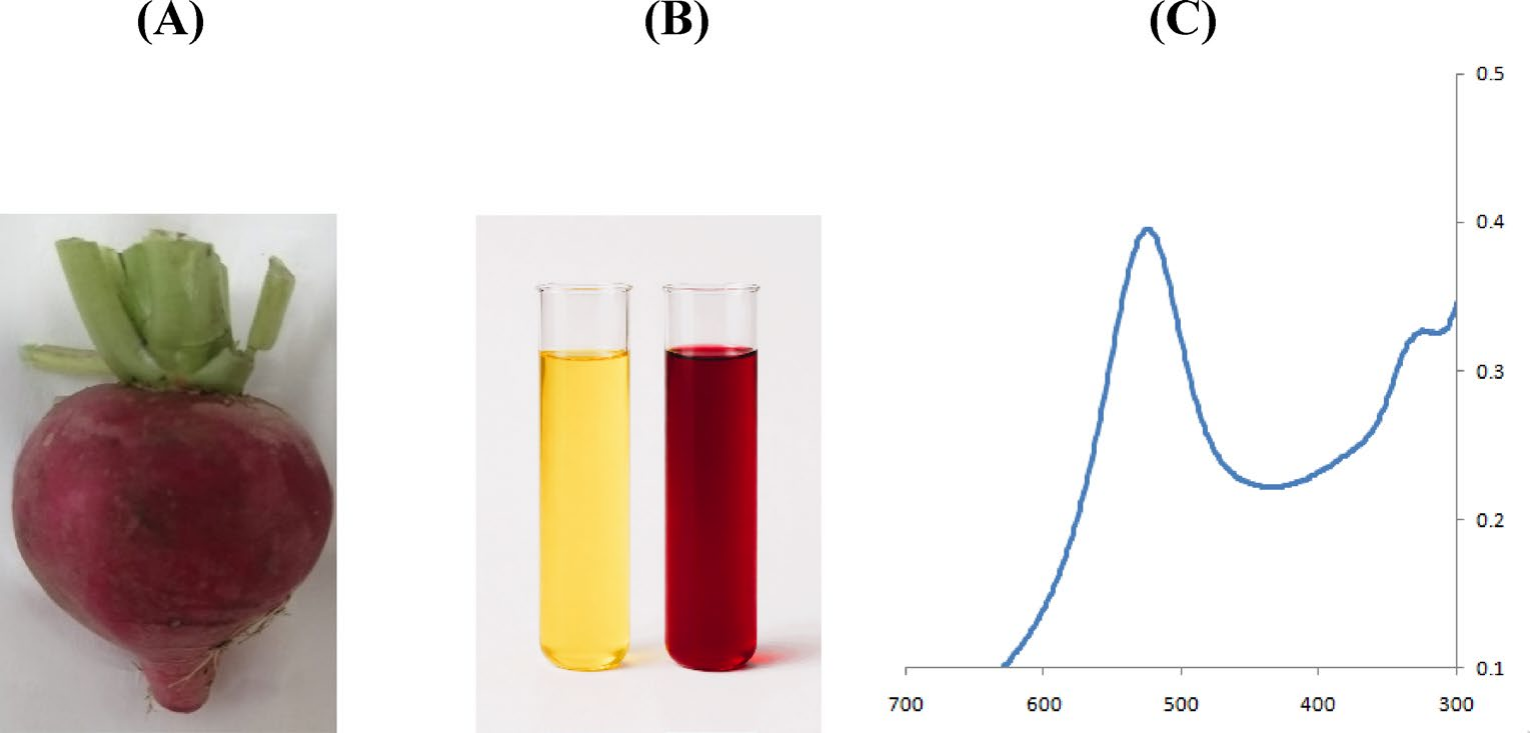
**(A)** Red Radish Root **(B)** Yellow gold ion solution (HAuCl4) and Ruby red RRR−AuNPs, and **(C)** UV/Vis absorption peak of synthesized RRR−AuNPs using Red Radish Root aqueous extract.

The UV/Vis spectroscopy is an important method to determine the stability and synthesis of AuNPs. The reduction of Au^+ 3^ to Au^0^ and nanoparticles formation was confirmed by the change of the solution’s color from yellow to red (Fig. 3B). The UV/Vis spectrum in the 190–800 nm region displayed a distinctive broad band which indicated the formation of AuNPs at a peak at 526 nm (Fig. 3C). The absorption of the resultant red color is attributed to the resonance of the surface plasmon for the AuNPs.

### Transmission electron microscope (TEM)

**Fig. 4:**
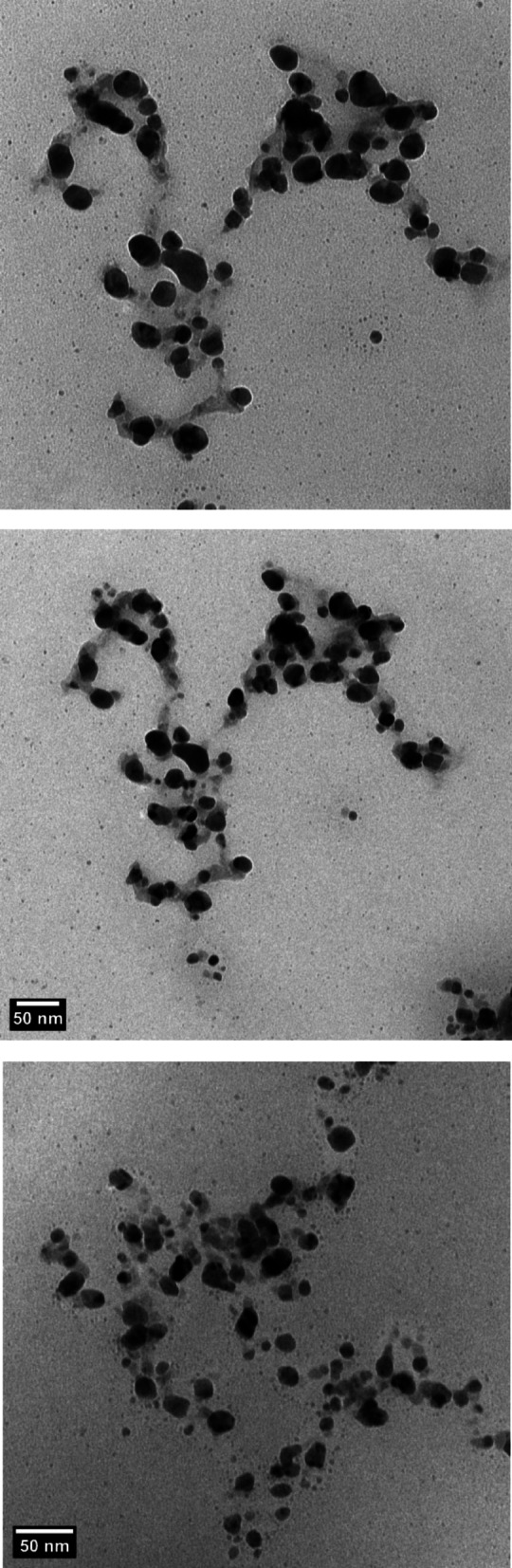
TEM image of RRR−AuNPs.

**Fig. 5:**
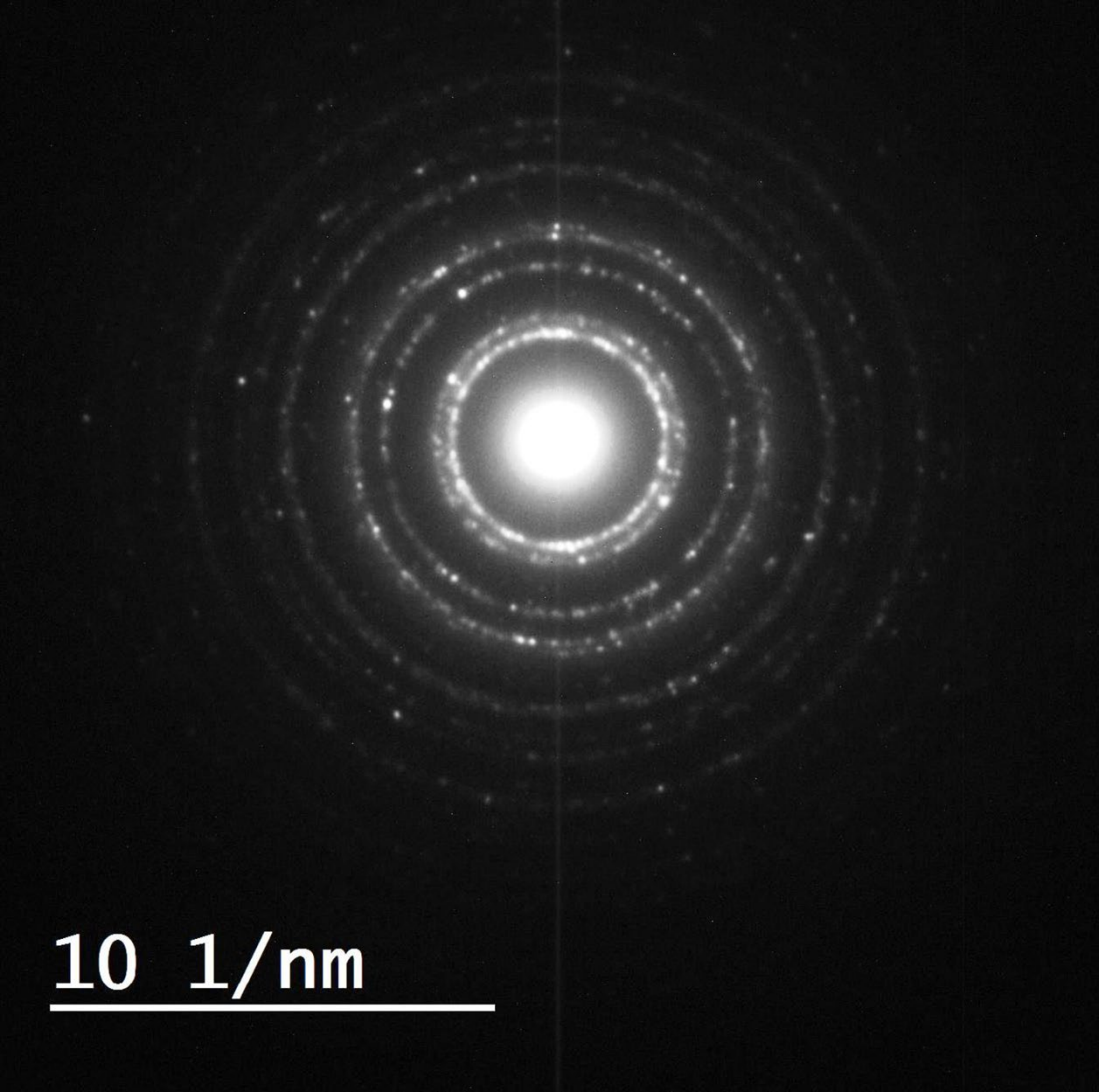
Selected area electron diffraction (SAED) analysis of the RRR−AuNPs. The sharp diffraction rings correspond to the (111), (200), (220), and (311) planes, establishing the characteristically face−centered cubic (fcc) metallic crystal phases of the nanogold.

Furthermore, the formation of RRR-AuNPs and analysis of their morphological characteristics was confirmed by Transmission Electron Microscopy (TEM). The TEM images obtained at various resolutions exhibited monodispersed spherical shapes of RRR-AuNPs with irregular morphologies Fig. 4. The selected area electron diffraction (SAED) result analysis showed the diffraction rings that could be indexed to the (111), (200), (220), and (311) crystallographic planes of face-centered cubic (fcc) metallic gold Fig. 5. These microstructural observations strongly corroborate the sharp diffraction peaks recorded in the XRD analysis, unequivocally confirming that the *Raphanus sativus* L. root extract successfully mediated the reduction of gold ions into highly crystalline metallic nanostructures.

**Fig. 6:**
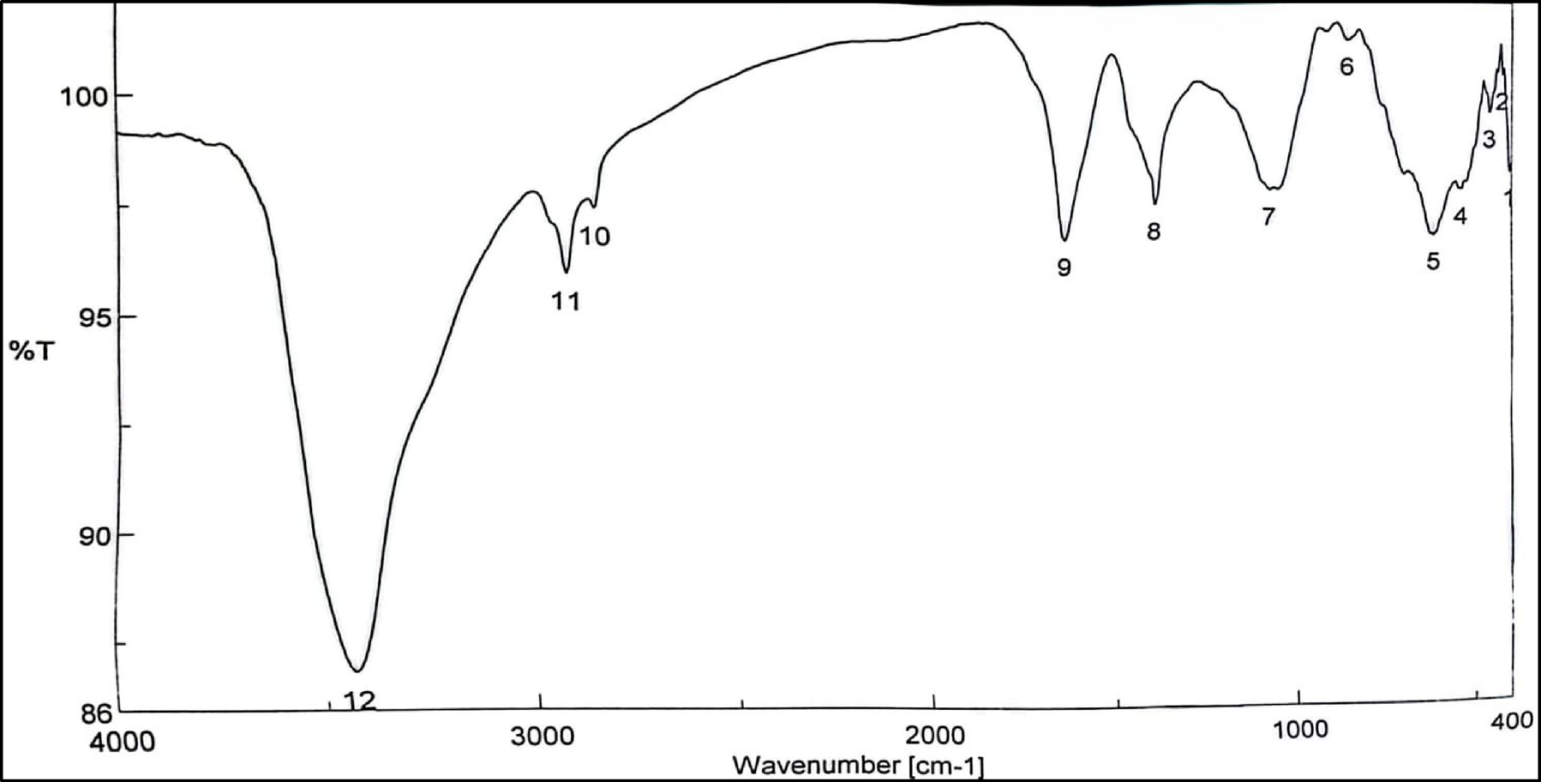
FT−IR spectra of RRR water extract.

**Fig. 7:**
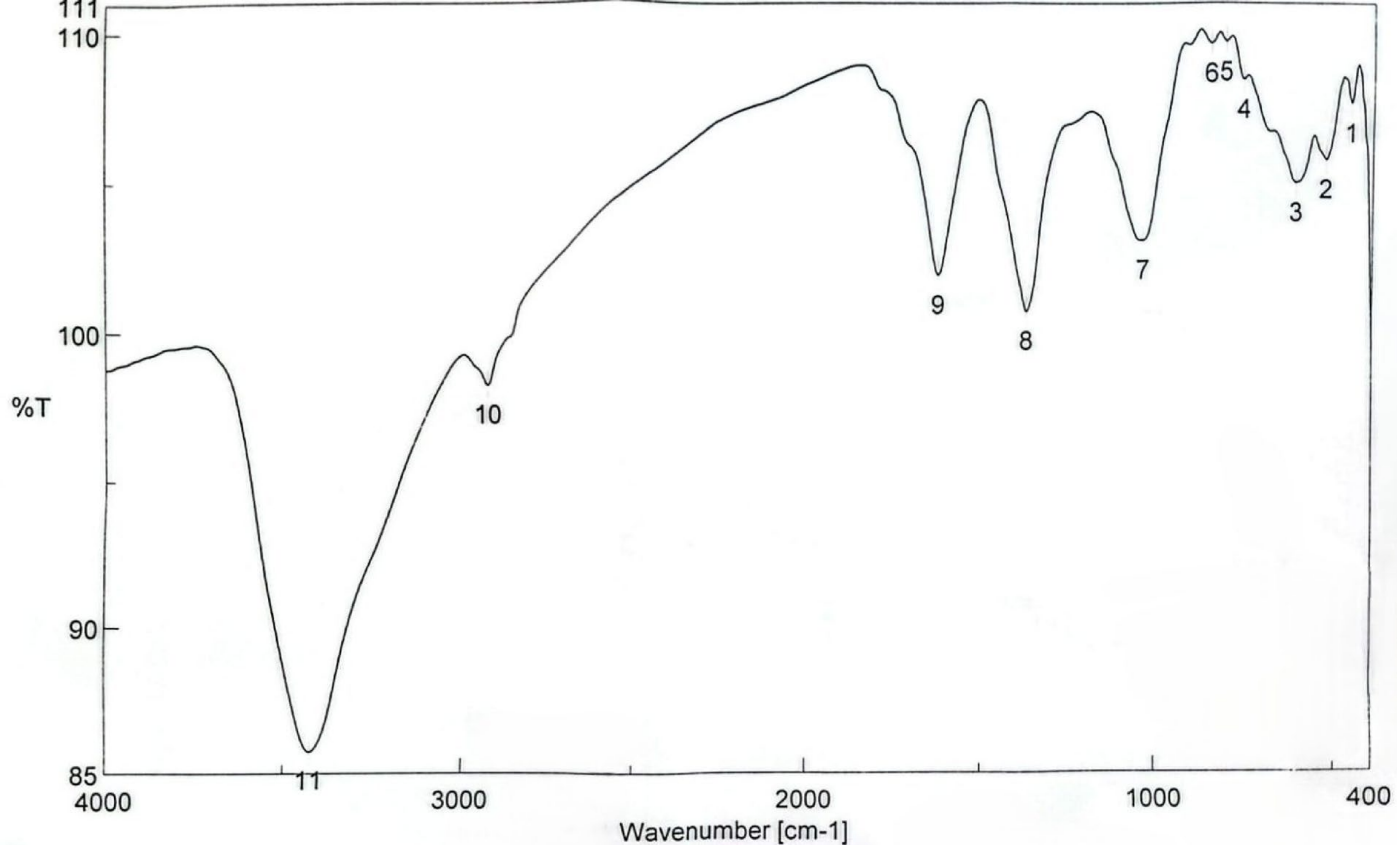
FT−IR spectra of RRR−AuNPs.

### Fourier transform infrared (FTIR) spectroscopy

The FT-IR spectra of the synthesized RRR-AuNPs and its extract were recorded in the % transmittance (%T) mode in the frequency range between 4000 and 400 cm^− 1^ Figs. 6,7. The analysis confirmed the interactions of gold nanoparticles and the functional groups of phytoconstituents of RRR water extract. Distinct vibrational bands for RRR-AuNPs were observed at 3426.89, 2924.52, 1628.59, 1381.25, 1060.66, 866.85, 824.42, 773.32, 620, 534.19 and 463.8 cm^− 1^. A strong, broad peak recorded at 3426.89 cm^− 1^ is attributed to O-H group stretching vibrations in RRR-AuNPs that shifted to 3431.71 cm^− 1^ in RRR water extract indicating the presence of alcohol and phenolic compounds. The C − H stretching band was observed at 2924.52 cm^− 1^ in RRR-AuNPs which shifted to doublet C − H at 2922.59 cm^− 1^ and 2854.13 cm^− 1^ in RRR. C − H bending band was identified at 1381.25 cm^− 1^ in RRR-AuNPs which shifted to1386.57 cm^− 1^ in RRR. The absorption band appearing at 1628.59 cm^− 1^ of RRR-AuNPs spectra corresponds to C = C stretching vibrations, confirming the presence of alkenes and aromatic structure.

Additionally, the C–O carbonyl stretching vibration appeared at 1060.66 cm^− 1^ in RRR-AuNPs which is shifted to 1077.05 in RRR. These diverse phytoconstituents, particularly polyphenols, alkaloids, and flavonoids, contain abundant hydroxyl O − H functional groups that facilitate the reduction of gold ions and the subsequent stabilization of the RRR-AuNPs.

**Fig. 8:**
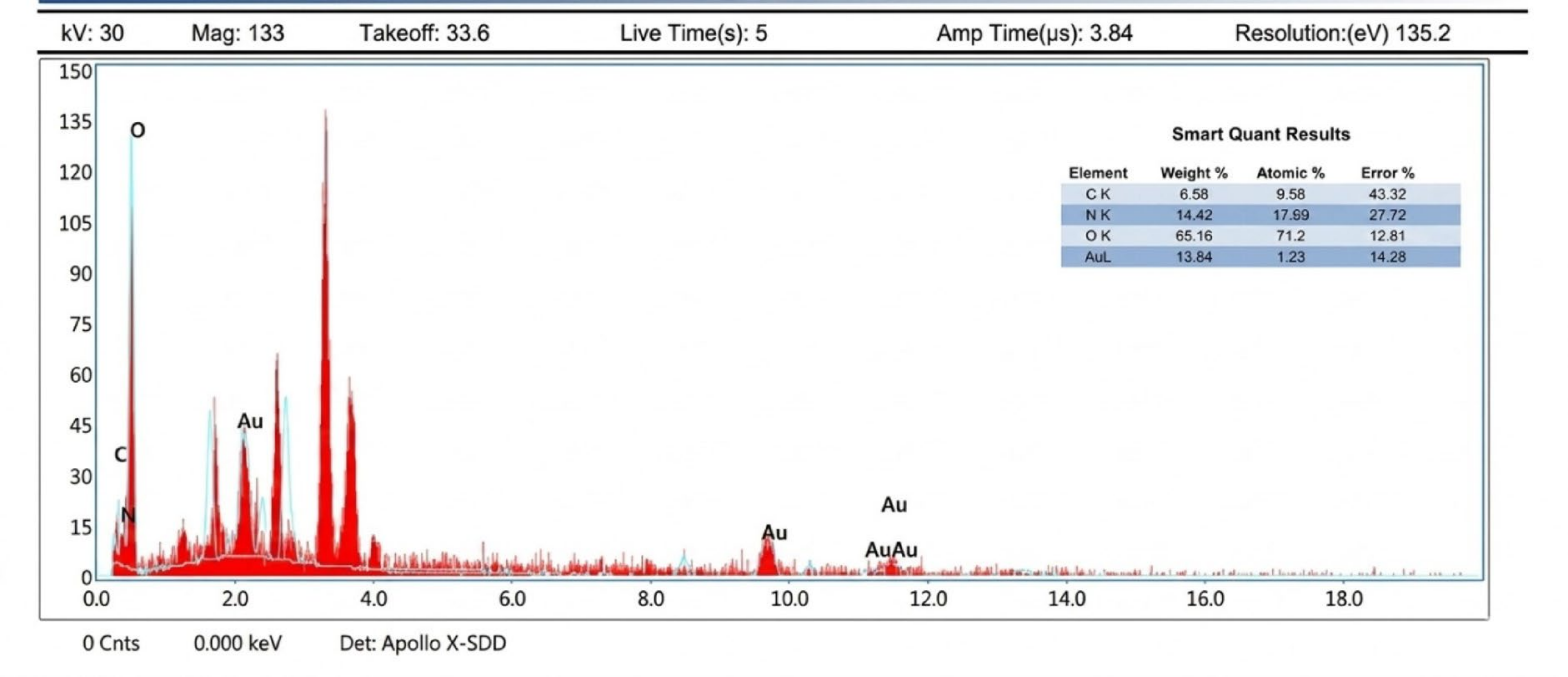
EDX pattern of AuNPs.

The EDX spectrum illustrated in Fig. 8 confirms the elemental profile of the synthesized RRR-AuNPs, where the prominent, intense peak validates the formation of highly pure metallic gold nanoparticles. Additional weaker signals corresponding to carbon, oxygen, and nitrogen which are attributed to the phytochemical compounds originating from the plant extract, confirming their role as capping and stabilizing agents adsorbed onto the nanogold surfaces.

### Atomic force microscope (AFM)

**Fig. 9:**
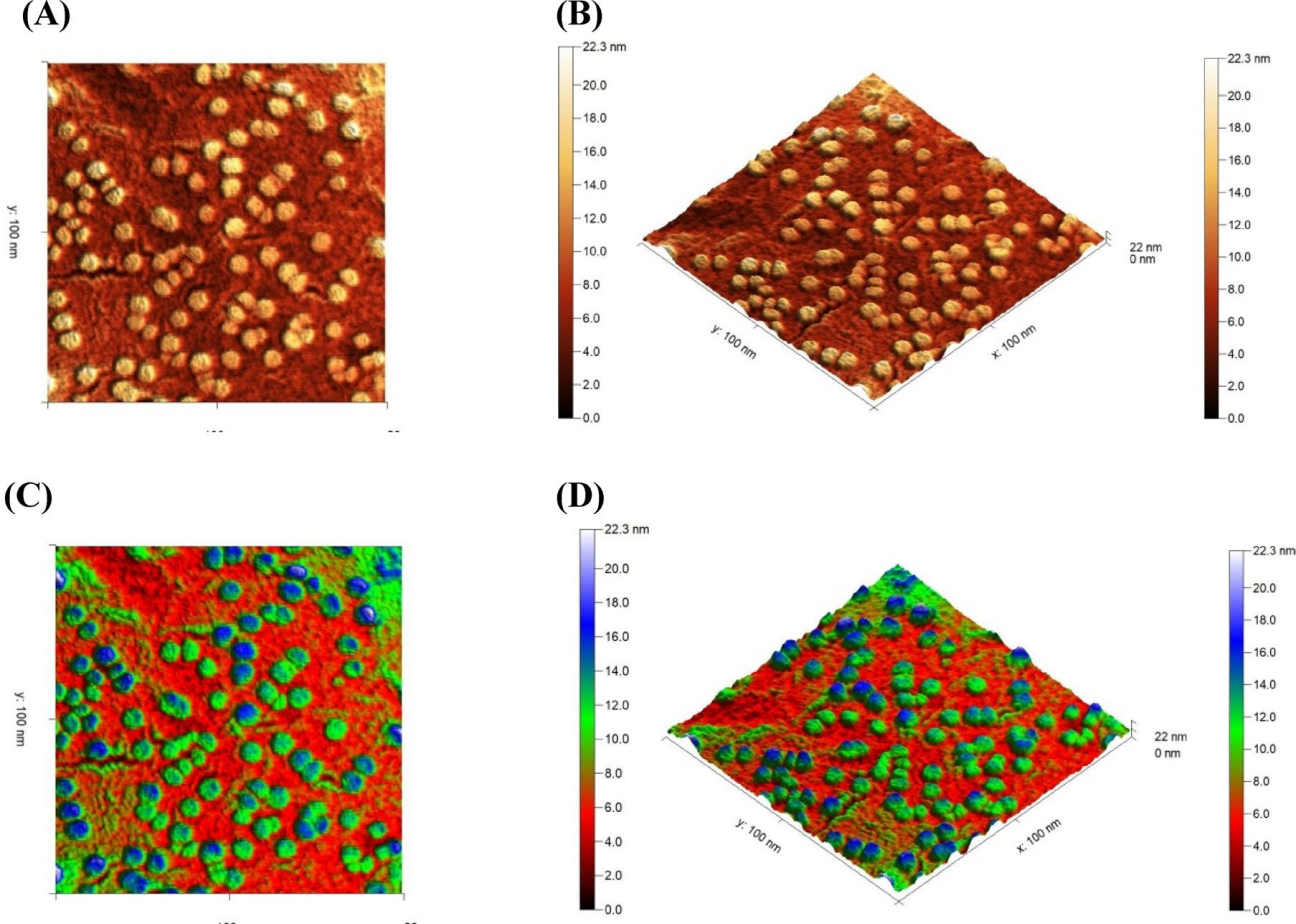
**(A)** top view (2D) AFM image of RRR−AuNPs; **(B)** 3D AFM image of RRR−AuNPs; **(C) t**op view (2D) AFM colored image of RRR−AuNPs, and **(D)** 3D AFM colored image of RRR−AuNPs.

Atomic force microscopic analysis is an advanced technique that was conducted to assess and confirm the shape (morphology) of RRR- AuNPs. AFM image illustrated the spherical shape of RRR- AuNPs with different particle size distribution (3D and 2D) Fig. 9. The current result was consistent with TEM and UV/Vis analysis and that prove the efficacy of red radish root extract as a reducing agent causing the spherical shape of RRR-AuNPs.

### Dynamic light scattering (DLS)

**Fig. 10:**
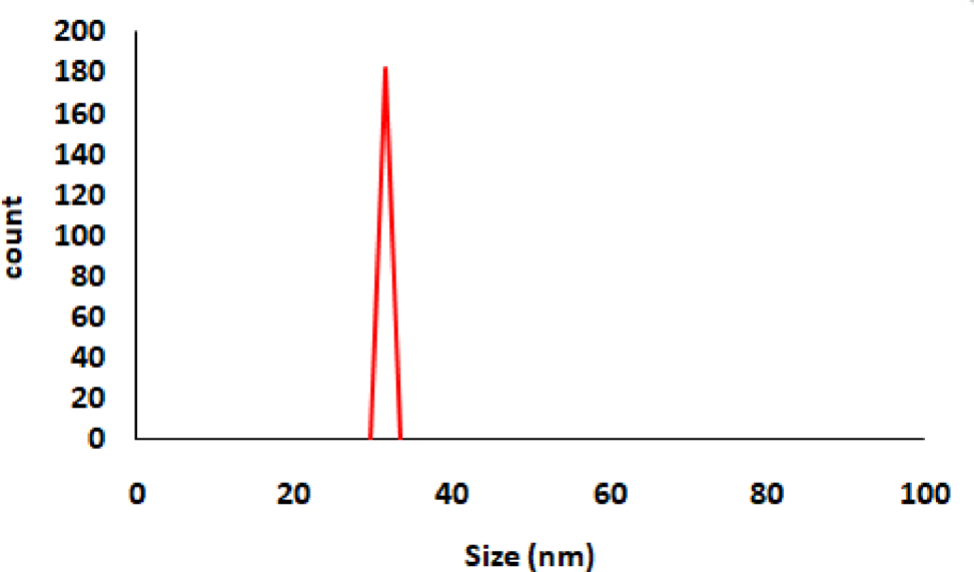
Particle size of RRR−AuNPs by Dynamic light scattering (DLS) analysis.

The DLS analysis revealed the composition of RRR-AuNPs and their uneven sizes, which matched the TEM results. Due to the varied content of RRR extract, the herbal extraction particles were not equal, which was correlated with the non-uniform size distribution of RRR-AuNPs. The main peak of the RRR-AuNPs curve, which represents the average size of the majority of particles, was distributed about 31 nm, according to DLS analysis. DLS that exhibited that particle size distribution was 29.5 and 33.6 nm Fig. 10.

### Zeta potential

**Fig. 11:**
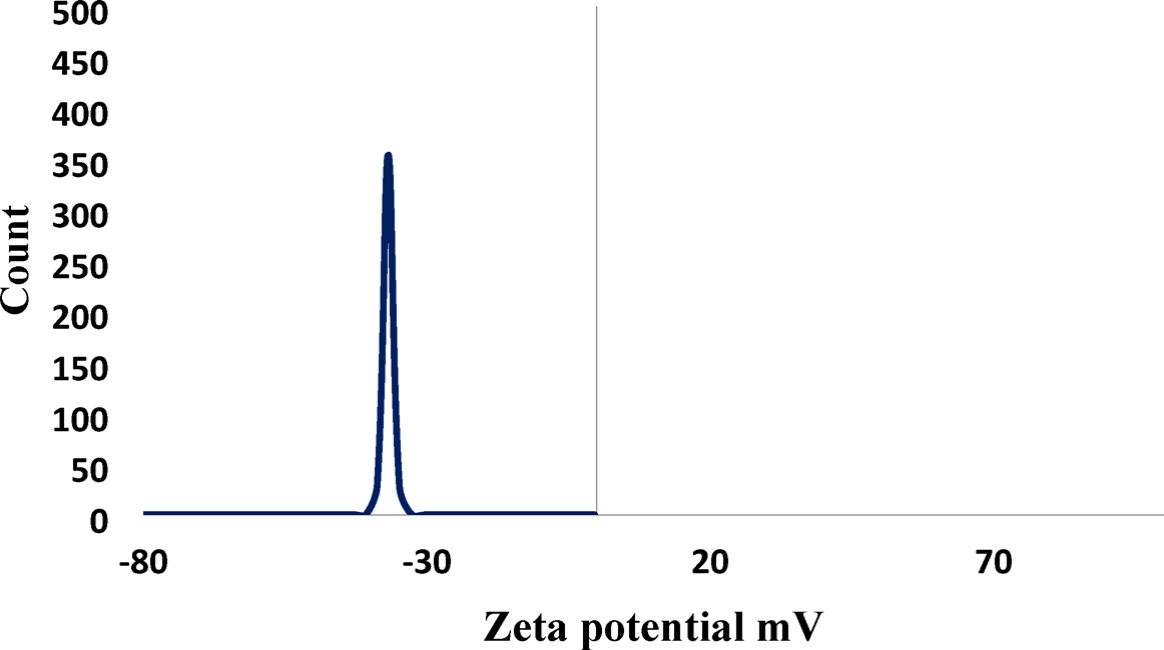
Zeta potential of RRR−AuNPs.

The zeta potential analyser was applied to detect the potential of surface on the particle at 25 °C. The Dynamic light scattering (DLS) was used to demonstrate the electrophoretic mobility of AuNPs. The generated zeta potential values of the formed nanoparticles were determined to be − 36.8mV. These values of zeta potential are considered as incipient stability of the obtained nanoparticles. The zeta potential for all the samples was measured by the particle size analyser Fig. 11. Particles with both smaller and larger size ranges can be detected in the TEM examinations.

### X-ray diffraction (XRD)

**Fig. 12:**
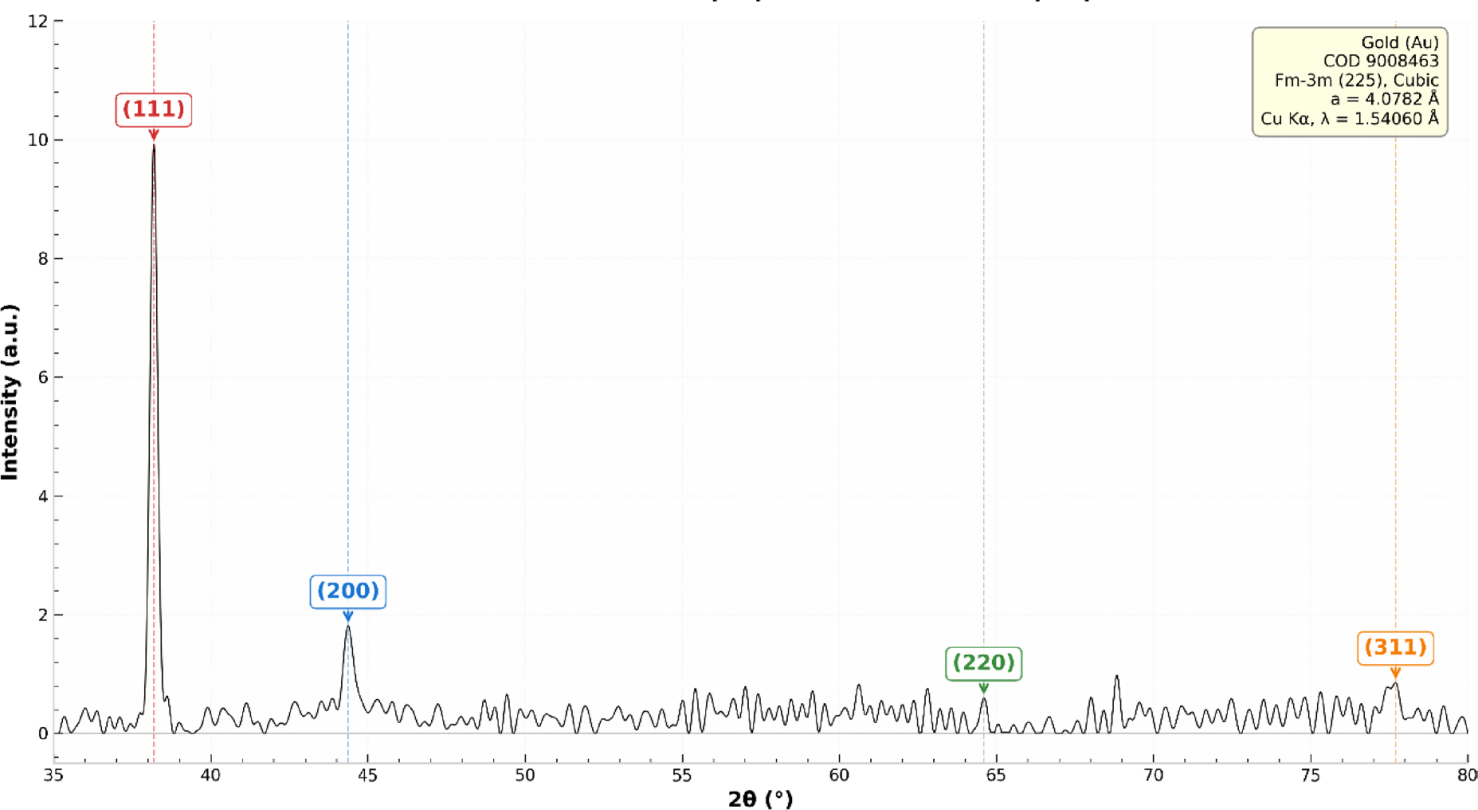
XRD pattern of RRR−AuNPs.

XRD analysis of the AuNPs crystalline phases is illustrated in Fig. 12. X-ray diffraction pattern of the sample confirmed the presence of gold in the AuNPs. The peaks for 2θ at 38.191^o^, 44.391^o^, 64.583^o^ and 77.679 ^o^ corresponding to the (111), (200), (220) and (311) planes of the face centred cubic (fcc) gold crystal, respectively. The sharp peak appears at 2θ = 38.010^o^ with 100% relative intensity showed most gold nanoparticles to be 31 nm in size.

### Surface area analysis

**Fig. 13:**
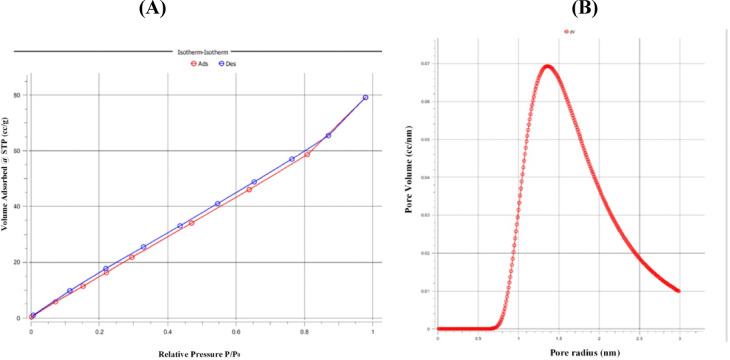
**(A)** isotherm curve and **(B)** DA curve of RRR−AuNPs.

The surface area estimated with the BET approach obviously showed how highly valued RRR- AuNPs. The average pore size was 3.24442 nm, total pore volume was 0.122832 cc/g and BET surface area was 75.7187 m²/g Fig. 13.

### Antimicrobial activity of RRR and RRR-AuNPs test (percent of reduction)

**Fig. 14:**
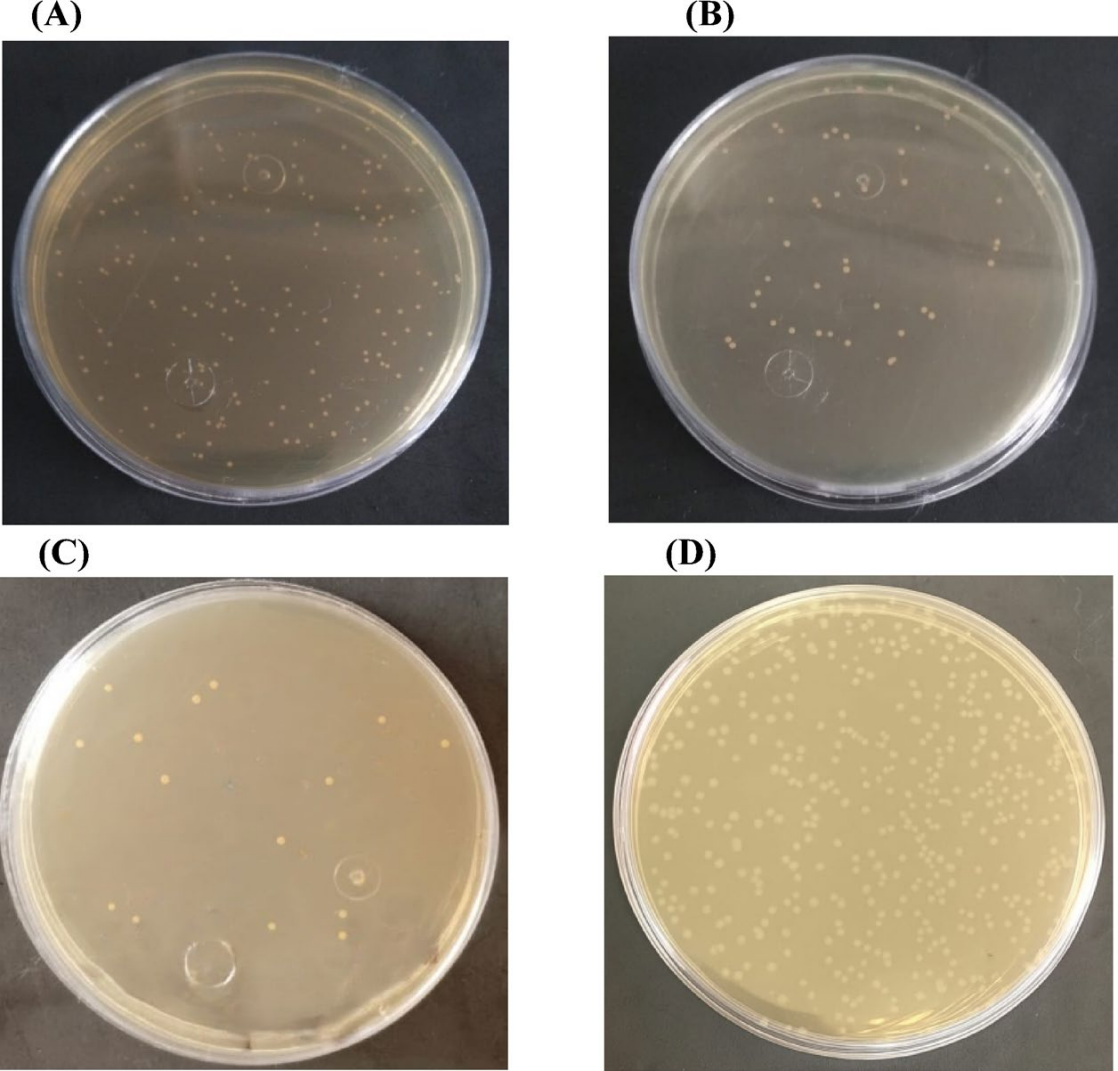
CFU count of growth inhibition test of *S. aureus* against **(A)** RRR extract **(B)** RRR−AuNPs, **(C)** Positive control (Amoxicillin) and **(D)** control sample (DMSO).

**Fig. 15:**
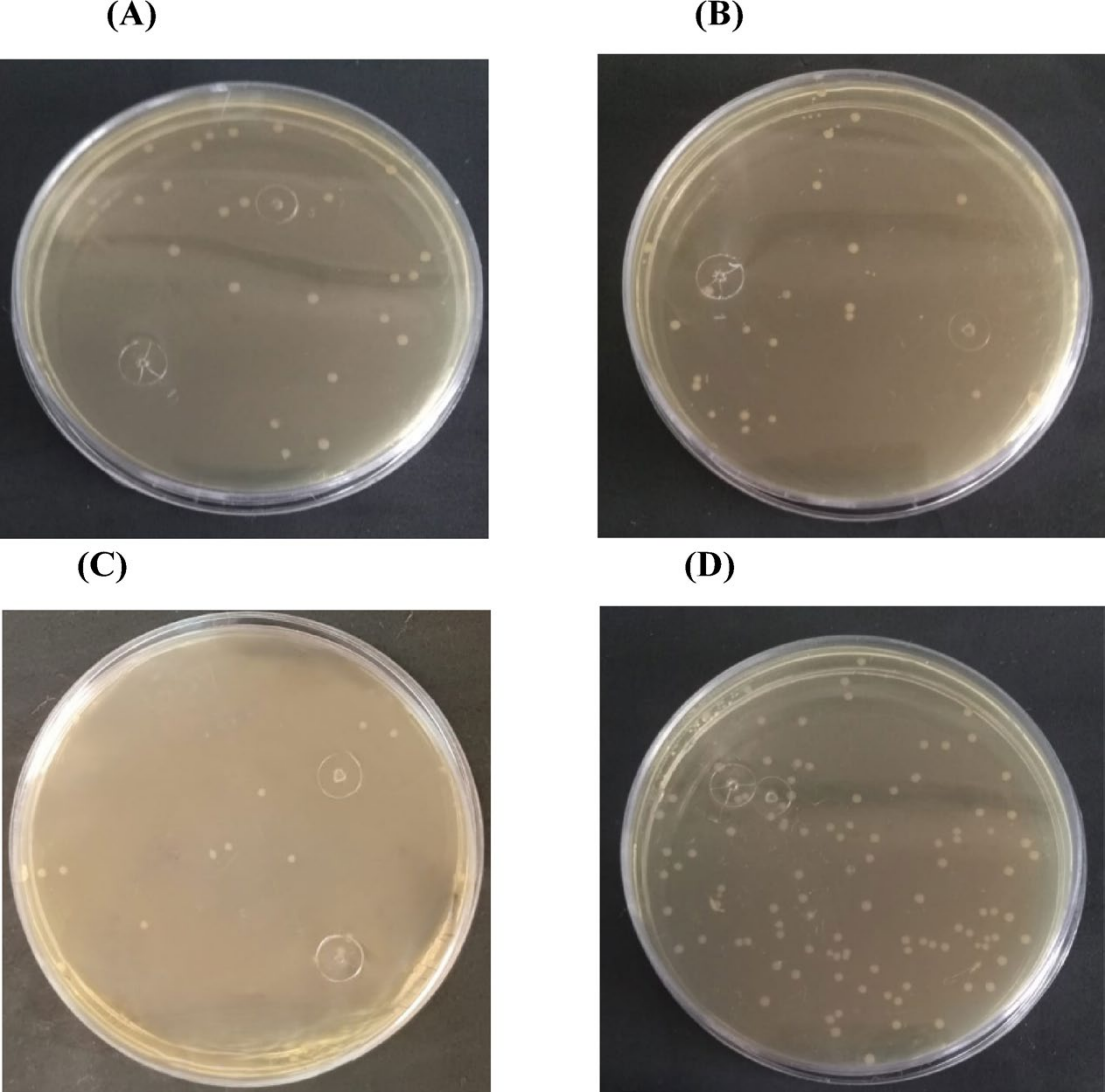
CFU count of growth inhibition test of *E. coli* against **(A)** RRR extract **(B)** RRR−AuNPs, **(C)** Positive control (Amoxicillin) and **(D)** and control sample (DMSO).

The antibacterial activity of RRR-AuNPs and RRR water extract was investigated using percent of reduction (inhibition) % test against *S. aureus* as a type of gram-positive bacteria (ATCC: 13565) and *E. coli* as a gram-negative one (ATCC: 10536). Plate counting test is used to evaluate the antibacterial activity of substances such as polymers and nanomaterials which have limited solubility. Colony forming unit counting for RRR-AuNPs revealed a highly inhibition percent of growth of *S. aureus* and *E. coli* with percent 80.9% and 78.2%, respectively (Tables 2, 3), Figs. 14, 15. Moreover, RRR water extract recorded a weak to moderate reduction percent of growth of *S. aureus* and *E. coli* with percent 27% and 67.3%, respectively (Tables 2, 3), Figs. 14, 15.

**Table 2:** Percent of Inhibition of growth of S. aureus treated with RRR and RRR-AuNPs.

S. aureus	RRR	RRR-AuNPs	Amoxicillin	Control	*p* value
Dilution Factor	10^− 3^	10^− 3^	10^− 3^	10^− 3^	
Volume of broth plated (µL)	20 µL	20 µL	20 µL	20 µL	
CFU at the dilution factor	168 ± 6.25^a^	44 ± 3.0^b^	15 ± 1.0^c^	230 ± 5.57^d^	0.000
Total CFU/mL	6,300,000 ± 234,187^a^	1,650,000 ± 75,000^b^	562,500 ± 37,500^c^	8,625,000 ± 208,791^d^	0.000
Log total CFU	6.80	6.22	5.75	6.94	
Inhibition %	27%	80.9%	93.5%	–	0.000

*CFU colony forming unit.*Data are represented as Mean±SD. Values that share the same letter at the same row are not significant. Values that share different letters at the same row are significant.

**Table 3:** Percent of Inhibition of growth of ***E. coli*** treated with RRR and RRR-AuNPs.

E.coli	RRR	RRR-AuNPs	Amoxicillin	Control	*p* value
Dilution Factor	10^− 4^	10^− 4^	10^− 3^	10^− 4^	
Volume of broth plated (µl)	20 µL	20 µL	20 µL	20 µL	
CFU at the dilution factor	36 ± 3.0^a^	24 ± 4.0^b^	9 ± 2.0^c^	110 ± 5.57^d^	0.000
Total CFU/mL	1,800,000 ± 150,000 ^a^	1,200,000 ± 200,000 ^b^	337,500 ± 75,000^c^	5,500,000 ± 278,388^d^	0.000
Log total CFU	6.26	6.08	5.53	6.74	
Inhibition %	67.3%	78.2%	93.9%	–	0.000

*CFU colony forming unit.*Data are represented as Mean±SD. Values that share the same letter at the same row are not significant. Values that share different letters at the same row are significant.

### MIC and MBC assays

The *Raphanus sativus* L. root (RRR) water extract demonstrated notable inhibitory effects against both *S. aureus* and E. *coli*, displaying a higher potency against the Gram-positive strain. In particular, the RRR water extract exhibited MIC values of 1.25 mg/mL against *S. aureus* and *E. coli* (Table 4). In addition, RRR water extract exhibited higher values of MBC (5 mg/mL).

In contrast, the *Raphanus sativus* L. root-mediated gold nanoparticles (RRR-AuNPs) exhibited substantial antibacterial efficacy compared to the crude water extract. Specifically, the RRR-AuNPs demonstrated MIC values of 0.977 µg/mL against *S. aureus* and E. *coli*. Moreover, RRR-AuNPs demonstrated lower MBC value of 15.63 µg/mL against the same tested strains as compared to the crude RRR extract (Table 4). Amoxicillin was utilized as a positive control, displaying an MIC value of 1.953 µg/mL and a corresponding MBC of 15.63 µg/mL, against *S. aureus* and *E. coli*. Remarkably, the biosynthesized RRR-AuNPs exhibited a comparable antibacterial profile, highlighting the exceptional potency of this green-synthesized nanomaterial as a highly competitive antimicrobial agent.

**Table 4:** Minimum inhibitory and bactericidal concentration of RRR water extract and RRR-AuNPs.

Sample	S. aureus MIC MBC	E.coli MIC MBC
RRR water extract (mg/mL)	1.25 5	1.25 5
RRR-AuNPs (µg/mL)	0.977 15.63	0.977 15.63
Amoxicillin (Positive control, µg/mL)	1.953 15.63	1.953 15.63

### Time killing assay

**Fig. 16:**
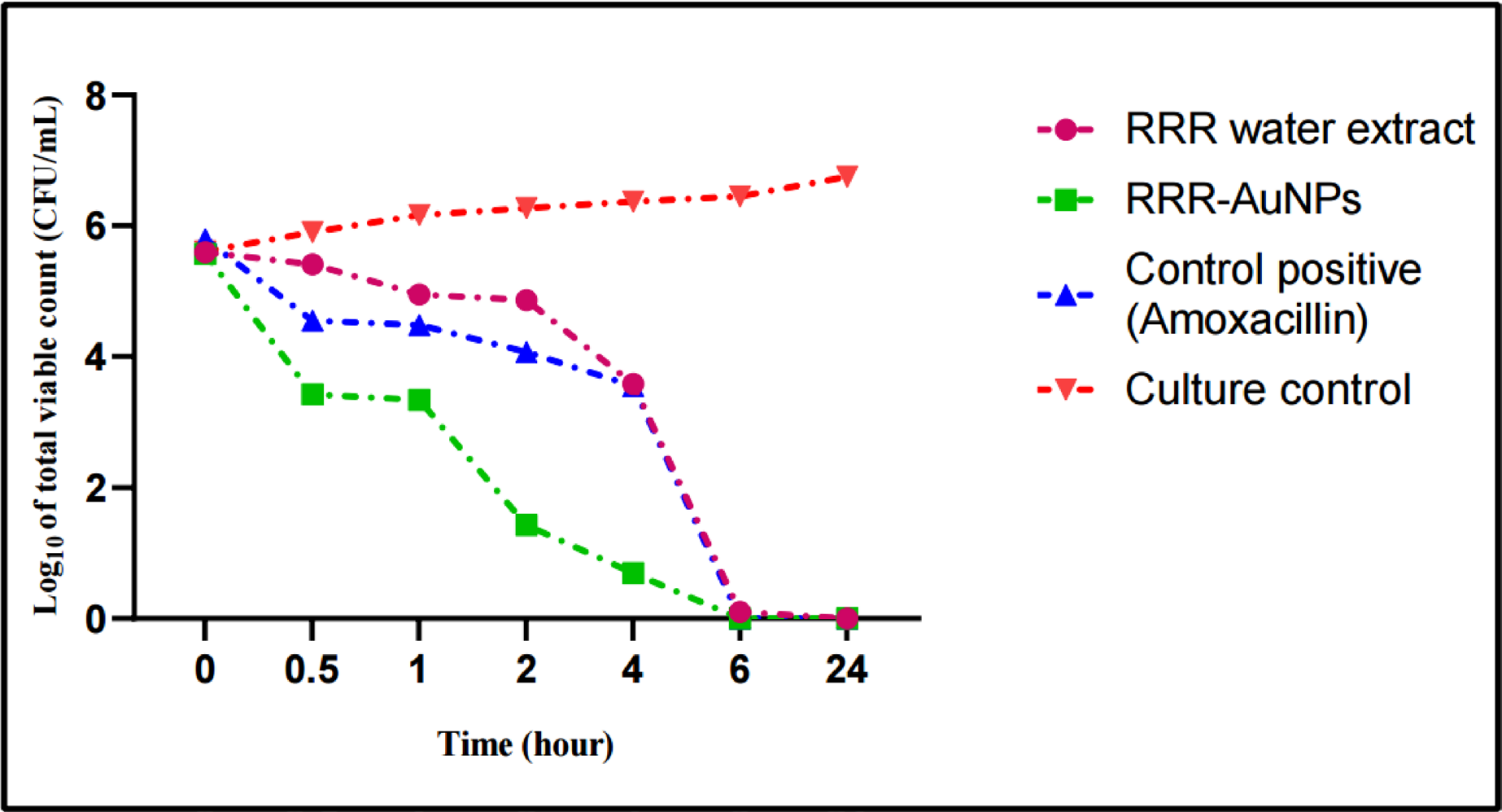
Rate of killing *Staphylococcus aureus* by RRR water extract and RRR-AuNPs.

**Fig. 17:**
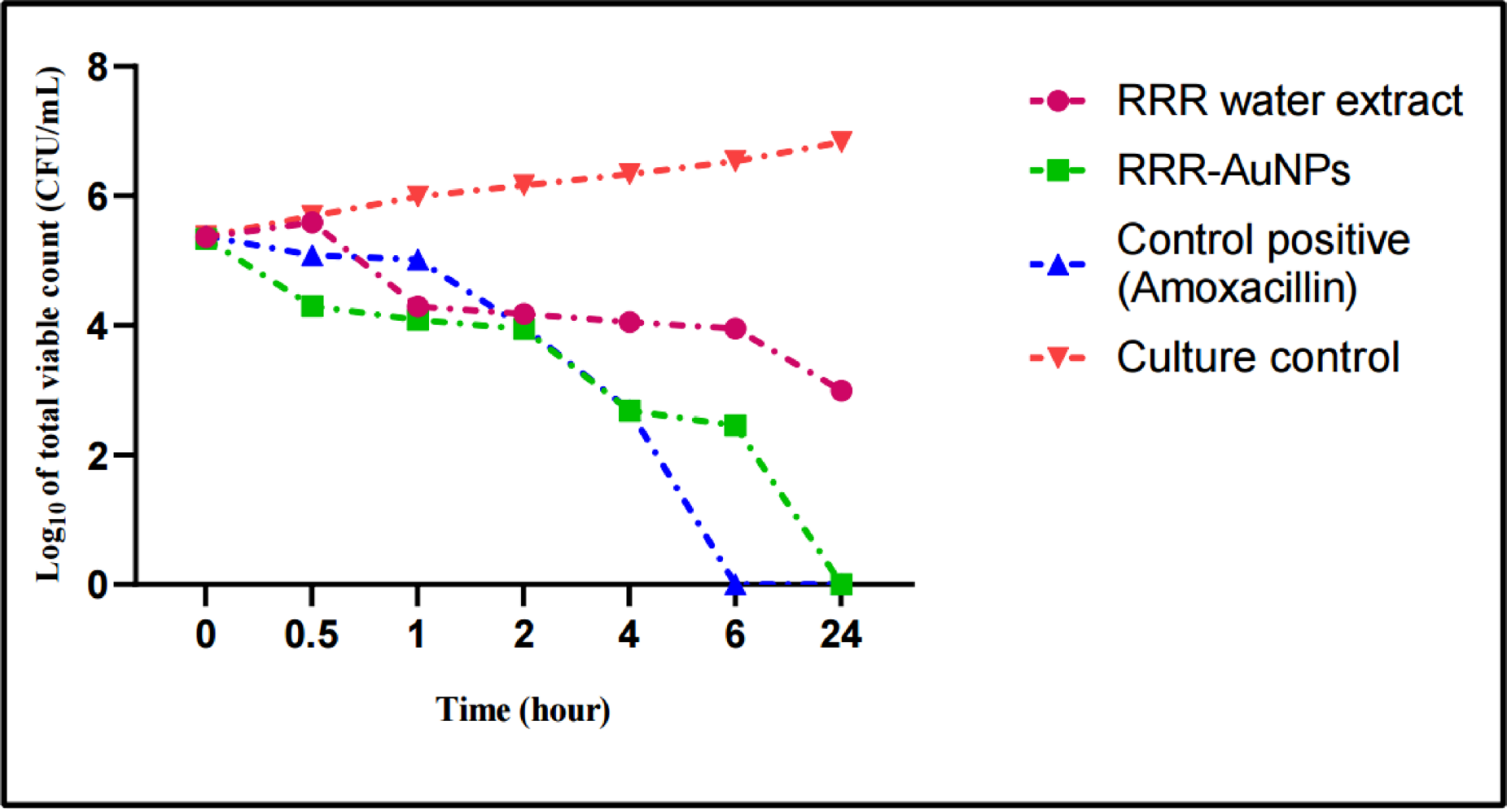
Rate of killing *E. coli* by RRR water extract and RRR-AuNPs.

The bactericidal profiles of the RRR water extract and RRR-AuNPs against *S. aureus* and *E. coli* were illustrated via time-kill analysis Figs. 16,17, respectively. Normal bacterial viability and proliferation were confirmed by negative control groups. Specifically, the untreated control populations exhibited continuous growth over the 24-hour incubation period, increasing from 5.61 log_10_ at 0 h to a final concentration of 6.75 log_10_ at 24 h while *E. coli* proliferated from 5.38 log_10_ at 0 h to 6.83 log_10_ at 24 h.

The time-kill dynamics curves revealed that the small-sized Au-NPs possessed exceptionally potent bactericidal activity. As illustrated in Figs. 16,17, complete eradication of *S. aureus* and *E. coli* occurred within 6 and 24 h of incubation with Au-NPs, respectively. In comparison, the crude RRR water extract exhibited slower kinetics, requiring a full 24 h to completely inhibit *S. aureus* and achieving only a partial reduction, rather than total clearance, of *E. coli* over the same duration (2.99 log_10_ at 24 h).

### Cell viability assay

**Fig. 18:**
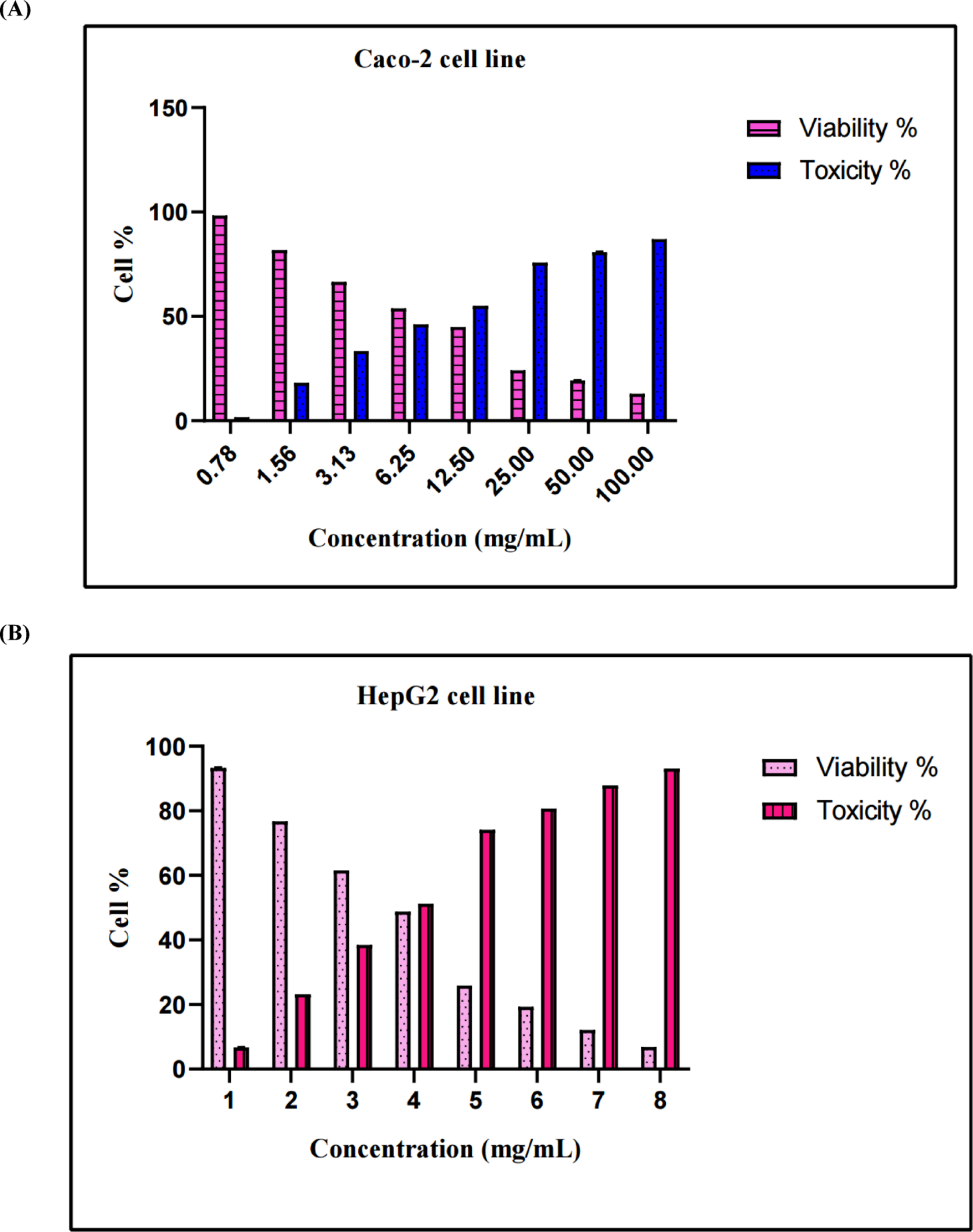
Cytotoxic effect of RRR extract against **(A)** Caco-2 colon and **(B)** HepG2 hepatic cancer cell lines.

MTT assay was performed to assess the cell viability and toxicity after treatment with different concentrations of RRR water extract (0.78, 1.56, 3.13, 6.25, 12.5, 25, 50 and 100 mg/mL) and RRR-AuNPs (31.25, 62.5, 125, 250, 500 and 1000 µg/mL) on colorectal adenocarcinoma (Caco-2) and hepatocellular carcinoma (HepG2) cell lines. The findings demonstrated that in both kinds of cancer cell lines, raising the dose considerably reduced cell viability. It was found that HepG2 and Caco-2 cell lines treated with RRR at 6.25 mg/mL reported cell cytotoxicity percent of about 51.21% and 46.22%. IC_50_ of RRR water extract against HepG2 and Caco-2 cell lines recorded 5.952 and 6.763 mg/mL, respectively Figs. 18 and 20.

**Fig. 19:**
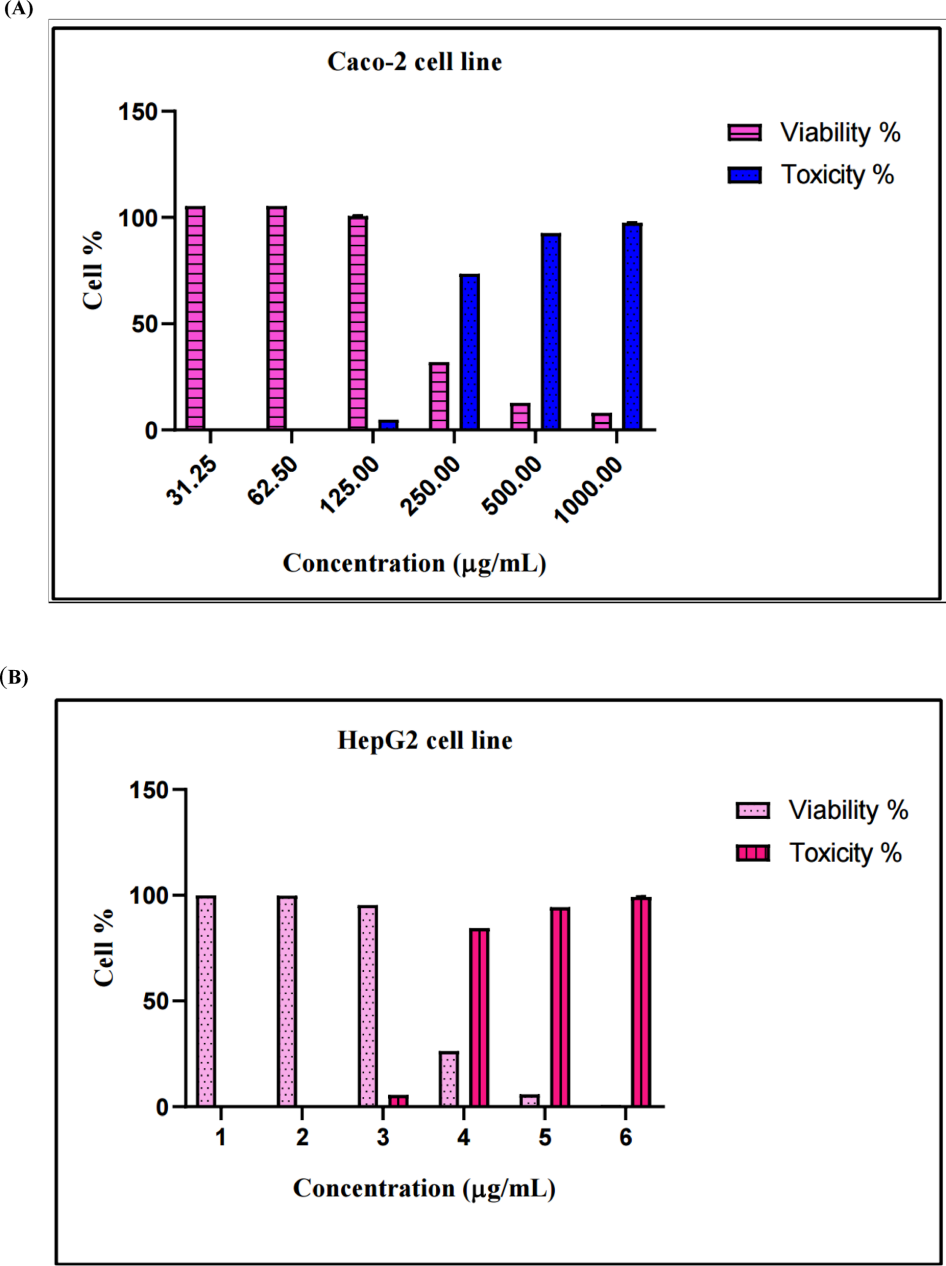
Cytotoxic effect of RRR-AuNPs against **(A)** Caco-2 colon and **(B)** HepG2 hepatic cancer cell lines.

**Fig. 20:**
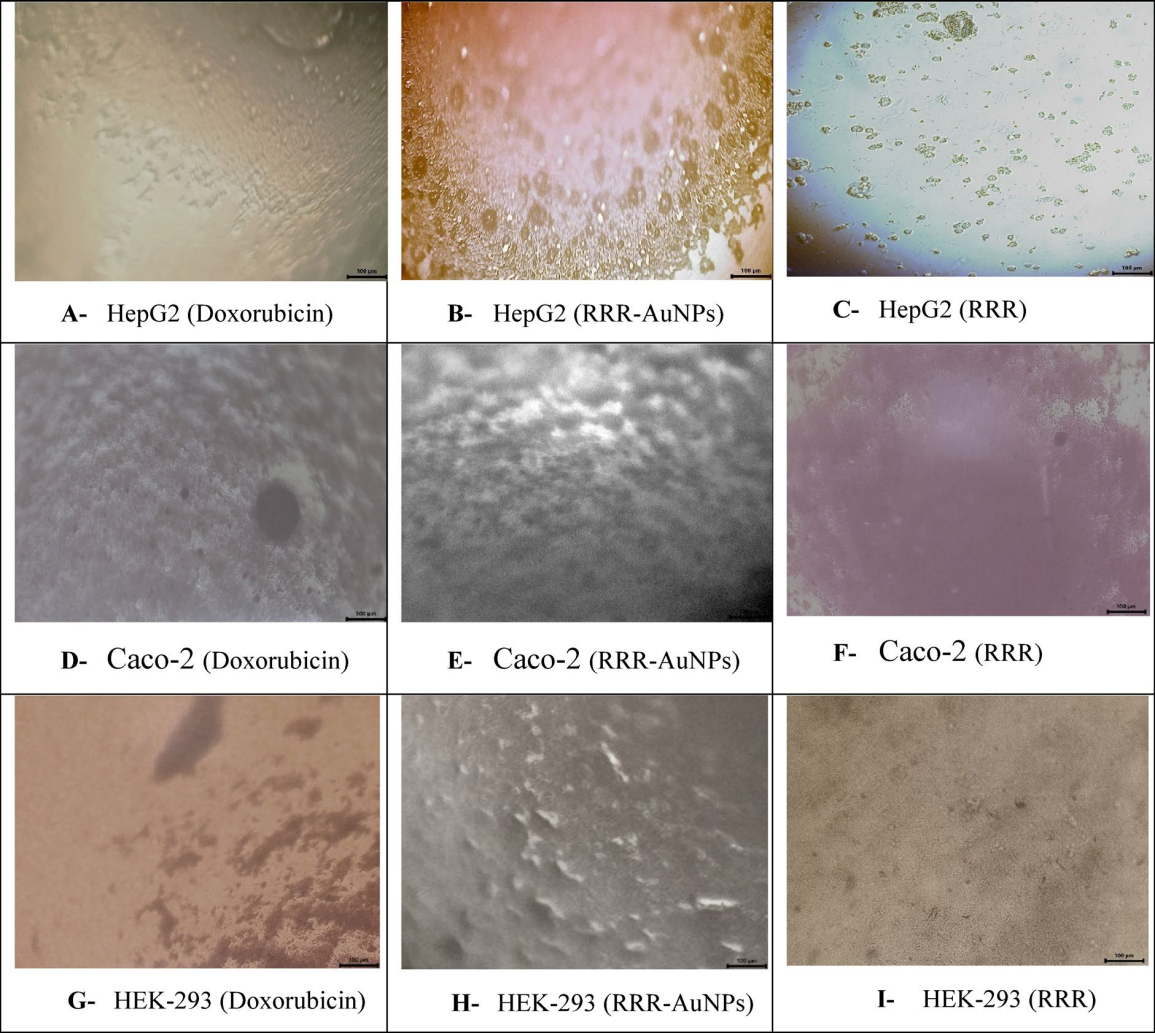
Representative cytotoxic activity images of RRR water extract (RRR), biosynthesized gold nanoparticles RRR-AuNPs (RRR-AuNPs) and Doxorubicin against colorectal adenocarcinoma (Caco-2), hepatocellular carcinoma (HepG2) and normal human embryonic kidney (HEK-293) normal cell line. Scale bar=100 μm (40x magnification).

Moreover, Caco-2 and HepG2 cells lines treated with RRR-AuNPs at 250 µg/mL recorded cell toxicity percent 73.6% and 84.4%, respectively confirming its cytotoxic effect against cancer cells. RRR-AuNPs were more potent in inhibiting hepatocellular carcinoma cell lines (IC_50_: 207.1173 ± 1.1 µg/mL) as compared to colon cell lines (IC_50_: 217.1875 ± 0.54 µg/mL) proving its effective biocompatibility Figs. 19, 20.

The safety profile of the biosynthesized RRR-AuNPs and the RRR water extract was evaluated against the HEK-293 (human embryonic kidney) normal cell line to assess their biocompatibility. The RRR water extract exhibited an IC_50_ of 12.16 mg/mL. In contrast, the synthesized RRR-AuNPs displayed an IC_50_ value of 296.07 µg/mL (Table 5). These results indicate that the gold nanoparticles possess a favourable safety margin on non-cancerous cells Fig. 20.

**Table 5:** IC50 of normal human embryonic kidney (HEK-293), MIC and selectivity index of RRR water extract and RRR—AuNPs against ***S. aureus*** and ***E. coli***.

Samples bacteria	RRR MIC (_mg/mL)_ IC_50 (mg/mL)_ SI	RRR-AuNPs MIC _(µg/mL)_ IC_50 (µg/mL)_ SI
S. aureus	1.25 12.16 9.73	0.977 296.07 303.04
E. coli	1.25 12.16 9.73	0.977 296.07 303.04

-Very high SI (SI≥100), High SI (SI between 10–99), Low SI (SI between1–9), Very low SI (SI<1)

The toxicological profile of the RRR water extract and the biosynthesized gold nanoparticles RRR-AuNPs relative to their antimicrobial potency were quantified using the selectivity index (SI). Based on the IC_50_ value of RRR-AuNPs obtained from HEK-293 normal cells (296.07 µg/mL) and the MIC values recorded for both *S. aureus* and *E. coli* (0.977 µg/mL), the RRR-AuNPs demonstrated a remarkable SI of 303.04. This high SI value indicates that nanoparticles are over 300 times more selective toward bacterial pathogens than human kidney cells. In comparison, RRR water extract exhibited IC_50_ of 12.16 mg/mL and an MIC value of 1.25 mg/mL for both strains, resulting in a substantially lower SI value of 9.73. These results highlight the superior biocompatibility and targeted antimicrobial efficacy of the synthesized nanoparticles over the crude extract.

## Discussion

The special phytochemical composition of RRR water extract causes the formation of the envelope surrounding gold metal atom and acts as a capping and stabilizing layer that prevents the aggregation of gold nanoparticles and the formation of larger structures. This result is in agreement with Timoszyk & Grochowalska, (2022)^30^.

Phytochemical compounds have an essential and defensive role against the harmful effects of free radicals and oxidative stress on the constituents of biological systems including lipids, carbohydrates and proteins biomolecules which can oxidize them resulting in cellular and tissue damage and many other diseases including inflammation, degenerative diseases and cancer^30^.

HPLC analysis of the RRR water extract revealed a complex profile of bioactive polyphenols such as catechins, gallic acid, caffeic acid, and chlorogenic acid, which constitute the primary phytoconstituents. Additional metabolites were detected including syringic acid, rutin, naringenin, vanillin, rosmarinic acid, ferulic acid, hesperetin, quercetin, coumaric acid, cinnamic acid, kaempferol, daidzein, and methyl gallate which assures the extract’s chemical diversity. These hydroxyl-rich polyphenolic compounds are of critical importance in this study, as they serve a dual role: acting as potent reducing agents for the transformation of gold ions into Au^0^ nanoparticles and subsequently functioning as capping ligands that stabilize the RRR-AuNPs. This diverse antioxidant assembly not only facilitates the green synthesis process but also synergistically enhances the antimicrobial and radical scavenging performance of the resulting biogenic nanomaterials. These findings are in alignment with Goyeneche et al., (2015)^31^.

The water extract of the roots of *R. sativus* has been screened for its DPPH free radical scavenging and reducing property, where the phytochemical components’ capacity that neutralise highly reactive free radicals was the main cause of the antioxidant property^30^. A relatively high antioxidant activity was obtained in this study for both RRR water extract and RRR-AuNPs with the higher value was for the RRR-AuNPs. Data obtained in this study is coincided with Noman et al., (2021)^32^ which suggested that RRR-AuNPs possess the ability to scavenge free radicals representing a powerful antioxidant, anti-inflammatory, anti-mutagenic and anticancer agent and might explain its anticancer activity towards HeP2G and Caco-2 cell lines that will be discussed later.

Furthermore, the antagonizing effect of polyphenols against radicals results from their prooxidant power which prevents lipid per-oxidation. The oxidation of all o-dihydroxylated phenolic derivatives results in the production of reactive oxygen species (ROS)^33^.

The AuNPs antioxidant effect was found to be higher than the antioxidant activity of the plant extract individually because the antioxidant phytochemical compounds of RRR extract are adsorbed onto the nanoparticles active surface. The high surface area-to-volume ratio of a nanoparticle and its surface reaction may also affect the interaction and free radicals scavenging activity, revealing a higher antioxidant potency. These results are compatible with^32,34,35^.

Synthesized nanoparticles have broad-spectrum applications in biomedicine, cosmetics, coating and, packing etc. Each application depends on the even size, composition, shape and stability of the prepared nanoparticles. Medicinal plant is used recently to prepare nanoparticles due to its simplicity, eco-friendless and its valuable content of bioactive compounds which have a strong capacity to bio-reduce gold heavy metal ion (Au^+^) into a stable nanoparticles metal (Au^0^)^36^.

The RRR aqueous extract reduced HAuCl4 leading to the biosynthesis of gold nanoparticles (AuNPs) which was confirmed by the formation of ruby red color after 24 h. The plant extract phytoconstituents are acting as both stabilizing and reducing agents during the nanoparticles’ synthesis. This result is compatible with Madhumithra, et al., (2018)^37^ which confirmed the formation of gold nanoparticles by the water extract of shells of *Pistacia vera* plant.

UV/Vis spectroscopy is the primary technique to confirm the nanoparticles’ formation and stability in an aqueous solution. The UV/Vis spectroscopy for greenly synthesized RRR-AuNPs indicated that the AuNPs were formulated successfully by recording peaks in the specific region for gold nanoparticles. It was documented that UV/Vis spectra for the gold nanoparticles exhibited plasmon peak about 525–540 nm and it’s a regular characteristic of spherical shaped AuNPs that have a diameter ranged between 30 and 50 nm^38,39^.

Predominantly, the RRR-AuNPs spherical shapes as showed by the HRTEM indicate the polydispersity nature of the synthesized nanoparticles. This might be due to the presence of different phytochemical compounds (reducing agent) in RRR extract which coated AuNPs forming particles of uneven size. Bright diffraction rings shown in SAED pattern also confirmed the crystallographic phase of the biosynthesized RRR-AuNPs. These rings appeared from the innermost to the outermost boundary as the (111), (200), (220), and (311) reflections, which perfectly correspond to the face-centered cubic (fcc) crystal lattice of metallic gold^31^.

Zeta potential is the fundamental factor that manages electrostatic interactions in particle dispersions. The zeta sizer and potential of AuNPs capped with *R. sativus* root extract were 31 nm and − 36.8mV, respectively. The synthesized AuNPs were very stable when stored in refrigerator and the particles of the colloidal AuNPs solution were not aggregated for several months. The high stability of the colloidal AuNPs suspension can be explained by its very high negative zeta potential value^40^.

XRD revealed that the peaks for 2θ at 38.191^o^, 44.391^o^, 64.583^o^ and 77.679^o^ corresponding to the reflections of the planes (111), (200), (220) and (311) supported the bio-reduction of Au (III) to Au (0) by the effect of the phytochemical compounds present in the root of water extract of *R. sativus* and also emphasizes the crystalline shape of gold atom^41^.

The dynamic light scattering (DLS) screening confirmed the size of RRR-AuNPs and was corresponding to those of TEM which indicated their disparate sizes. The uneven size distribution of RRR gold nanoparticles was accounted for the diverse coating of AuNPs with various phytochemicals of *R. sativus* root extract^12^.

In FT-IR spectra, the presence of stretching bands of O-H for alcohols or phenols, C = C for alkenes or aromatics and C–O for carboxylic acids suggest that polyphenols and flavonoids were involved in the formulation of AuNPs through the reduction of gold ions to gold atoms. Phenolic compounds are documented to possess an exceptional binding affinity for metallic ions, which directly facilitates the reduction of gold ions to elemental gold while inducing a robust chelation effect. Concurrently, the carboxylate groups serve as natural surfactants during nanoparticles synthesis. The strong affinity of these carboxylate groups for the newly formed AuNPs inhibit nanoparticle aggregation and maintaining long-term colloidal stability^31^.

Energy-dispersive X-ray (EDX) spectroscopy is a widely utilized analytical technique for identifying the elemental composition and purity of synthesized nanomaterials. EDX characterizes the metallic core alongside any associated organic elements derived from the plant extract. The EDX spectrum of the synthesized RRR-AuNPs displayed a distinct, dominant absorption peak at approximately 2.2 keV, which is uniquely characteristic of metallic gold (AuL-alpha shell energy). The study of Barai et al., (2018) is compatible with our findings^41^.

AFM analysis is used to estimate the morphology of nanoparticles’ surface. AFM image proved the spherical shape and the rough surface of the gold nanoparticles capped with *R. sativus* root extract^12^. AFM has reported versatility in various fields of study representing a dynamic method for conceiving the topographic characteristics of nanoscale. The current findings revealed that the bioactive compounds that capped AuNPs were agglomerated resulting in the synthesis of discrete nanostructures. The dissimilar size and morphology of the AuNPs is attributed to the presence of both single and aggregated nanoparticles. These results are harmonized with Ullah et al., (2021)^42^.

The surface area measured by the BET method of N2 adsorption–desorption isotherm curves clarified the high surface area of green synthesized RRR-AuNPs. The adsorption isotherm curve of synthesized nanoparticles showed the V-type isotherm at which the effects of intermolecular attraction are great, and the adsorption occurs in pores and capillaries^43^. This indicates that RRR-AuNPs have a large distance of pores and capillaries.

The current findings reported that the synthesis of AuNPs using water extract of *R. sativus* root as a reducing agent had a potential antibacterial effect against *S. aureus* and *E. coli*. Bacterial colony forming unit counting test showed a high percent of inhibition of the growth of those two bacterial strains after 24 h of treatment by RRR-AuNPs which confirms the powerful antibacterial activity of gold nanoparticles capped with red radish root extract. The zone of inhibition test is unfavourable to be applied in nanoparticle solutions because such solutions are suspension which dispersion in the solidified agar media is imperfect causing inaccurate results; therefore, percent of inhibition test is used instead^44^.

The antibacterial effect of the gold nanoparticles depends on many factors such as the rate of absorption, metabolites release, metabolic functions and its distribution in the cell. The surface positive charge of gold nanoparticles binds and interacts greatly with the negative charge of surface of bacterial cell wall through active moieties penetrating the cell, where they start to interact with its different components. Hydrophobic AuNPs that have a positive surface charge, can create localized aggregates on the of bacterial cell surface leading to easy penetration of the cell wall. Moreover, the modulation of bacterial cell membrane and the electrostatic interactions were the fundamental mechanisms through which the AuNPs stimulated its antibacterial effect. The bacterial strain can also affect the mode of interaction with bacterial cells^30,42^.

In addition, RRR water extract showed a week to moderate inhibition activity of growth of *S. aureus* and *E. coli* and that was attributed to phytochemical constituents such as phenolics, flavonoids, and glucosinolates present in radish root extract can interact with bacterial cell membranes, increase membrane permeability, disrupt nutrient uptake and many cellular processes, leading to antibacterial effects. These findings are in accordance with Ziemlewska et al., (2024)^45^.

The minimum inhibitory and bactericidal concentration profiles of the crude RRR water extract and the biogenic RRR-AuNPs revealed distinct susceptibility patterns across the tested strains. The crude RRR water extract exhibited a more pronounced delay in neutralizing the Gram-negative bacteria as compared to the Gram-positive one, requiring substantially higher concentrations to achieve complete bactericidal eradication (MBC) than to merely inhibit vegetative growth (MIC). These findings align with previous literature, which often attributes the relative resistance of Gram-negative bacteria to their complex outer membrane structure. This lipid bilayer effectively acts as a selective permeability barrier, limiting the influx and penetration of various phytogenic antimicrobial agents. Conversely, the phytogenic AuNPs successfully bypassed these structural defences, demonstrating potent bacterial inhibition and eradication at low microgram thresholds. This superior antimicrobial potency can be attributed to the high surface area-to-volume ratio of the nanomaterials, which facilitates more effective interactions with the bacterial cell envelope. Furthermore, their nanoscale size range promotes efficient cellular uptake and penetration, while a synergistic effect between the surface-capped phytoconstituents and the gold core amplifies the overall bactericidal toxicity, as conclusively corroborated by the alignment of the MIC and MBC datasets.

Adegbola et al., (2026) successfully utilized onion peel and bulb to synthesize AuNPs that inhibited the growth of Gram-positive bacteria like *S. aureus* and *Streptococcus pyogenes* and Gram-negative bacteria like *E. coli* and *pseudomonas aeruginosa*^46^. Likewise, Nayem et al., (2020) documented that the biogenic synthesis of AuNPs and AgNPs using *Amorphophallus paeoniifolius* had notable antibacterial effects. This bactericidal performance is driven primarily by their nanoscale morphology and high specific surface area, allowing for enhanced cellular penetration and membrane interactions^47^.

Time-kill kinetic profiles revealed that green-synthesized RRR-AuNPs possessed significantly accelerated and superior bactericidal potency compared to the crude RRR water extract. Against *S. aureus*, the nanoparticles achieved complete eradication within 6 h, reducing viable counts to zero, whereas the RRR extract required a full 24 h for clearance. For Gram-negative *E. coli*, RRR-AuNPs systematically eliminated the population after 24 h. Conversely, the crude RRR water extract exhibited a more protracted and limited antimicrobial effect; while it successfully suppressed the population compared to the untreated control, a flexible bacterial fraction survived, leaving a residual load of 2.99 log_10_ CFU/mL.

The minimum inhibitory/bactericidal concentrations (MIC/MBC) time-kill profiles conclusively demonstrate the superior antibacterial efficacy of RRR-AuNPs compared to the crude red radish root extract. Driven by their nanoscale dimensions, high surface reactivity, and phytochemical capping layers, these biogenic nanoparticles readily attach to and penetrate the bacterial cell wall, inducing lethal damage to intracellular DNA and proteins. Ultimately, these findings confirm that sustainably synthesized RRR-AuNPs exert enhanced, broad-spectrum bactericidal activity against both Gram-positive and Gram-negative pathogens, representing promising candidates for advanced antimicrobial therapeutics. These results correspond with those of Adegbola et al. (2026), highlighting the potent and rapid antimicrobial action of gold nanoparticles synthesized via eco-friendly onion bulb and peel extracts^46^.

The MTT test is an in vitro model applied to estimate the cytotoxic impact of substances against various cancer cell lines. The current study suggested the use of RRR-AuNPs in hepatic and colorectal cancer. The treatment of Caco-2 and HepG2 cell lines with the AuNPs capped with *R. sativus* root extract suppressed the cell viability of cancerous cell highly significantly at 250 µg/mL concentration. These results are harmonized with Abel et al., (2016) and Zhao et al., (2021)^12,48^.

Moreover, the RRR water extract showed anticancer activity in Caco-2 and HepG2 cell lines and that may be attributed to its high content of glucosinolates, phenolics, flavonoids, isothiocyanates, sulforaphane and others which exhibit anticancer properties. These compounds can trigger apoptosis via mitochondrial pathways, modulate detoxifying enzymes (Phase I/II), arrest the cell cycle (particularly at G2/M phase), and inhibit proliferation and metastasis in various cancer types. Additionally, activation of cellular protective pathways such as Nrf2/AhR contributes to enhanced detoxification and antioxidant response, thereby suppressing tumorigenesis^49^.

The biocompatibility of the greenly synthesized RRR-AuNPs was critically evaluated against the HEK-293 normal human kidney cell line. In the current study, Although the RRR-AuNPs exhibited a lower IC₅₀ and higher relative cytotoxicity compared to the crude water extract, they maintained a remarkably wide therapeutic window. This was quantified by an antimicrobial selectivity index (SI) which was calculated as the ratio of the IC₅₀ on normal cells to the MIC against pathogenic bacteria. The RRR-AuNPs yielded an SI of approximately 303, indicating that the nanoparticles are over 300 times more toxic to bacteria than to human kidney cells. This high selectivity is likely attributed to the unique surface chemistry provided by the *Raphanus sativus* capping agents, which may enhance bacterial membrane interaction while remaining relatively inert toward mammalian cell membranes at therapeutic concentrations. Consequently, the high SI value highlights RRR-AuNPs as a highly biocompatible candidate for clinical applications, where potent antibacterial action is required without compromising host cell viability. This methodology is consistent with Belete et al. (2025) which utilize the SI to establish a therapeutic safety margin^29^. Our observed SI of 303 significantly exceeds the common safety threshold (SI > 2), reinforcing the potential of RRR-AuNPs as a non-toxic antimicrobial agent. Compared to the synthesized nanoparticles, the raw RRR water extract displayed a diminished SI value of 9.73. Although the extract maintains a favorable margin of safety (SI > 1), its therapeutic window is markedly narrower than that of its synthesized nanoparticle counterpart, highlighting the enhanced bioactivity achieved through green nanotechnology. Tiras et al., (2024)^50^ have previously demonstrated the antiproliferative activity of red radish methanolic extract on Human Embryonic Kidney cells (HEK293) cells with lower IC₅₀ values (160 mg/mL) indicating stronger cytotoxicity.

In the present work, it is suggested that RRR-AuNPs effectively target and infiltrate into tumour cells with higher bioavailability and loading efficiency, confirming its important role in drug delivery targeting into cells and effective release drug applications. Moreover, the significant disparity between the cytotoxic threshold (IC_50_) of the RRR-AuNPs and the effective therapeutic antibacterial and antioxidant concentrations demonstrates a favorable biocompatibility profile. Consequently, RRR-AuNPs represent a versatile and safe platform for future clinical and biomedical advancements.

The present study possesses several notable strengths, primarily the successful development of a completely green and rapid synthesis of gold nanoparticles using *Raphanus sativus* root extract, which acts as both a reducing and stabilizing agent without the need for toxic chemicals. A significant strength of this work is the establishment of an exceptionally wide therapeutic window, as evidenced by a Selectivity Index (SI) of 303.04. This demonstrates that the nanoparticles are over 300 times more toxic to bacterial pathogens than to HEK-293 normal cells. Furthermore, the RRR-AuNPs displayed potent, dose-dependent antioxidant and antibacterial activities, particularly against *S. aureus* and *E. coli*. However, some limitations must be acknowledged. Although the antimicrobial and cytotoxic profiles were clearly established in vitro, still further in vivo investigations are necessary to evaluate the systemic pharmacokinetics and long-term biocompatibility of these nanoparticles within complex biological systems. Additionally, while XRD and FT-IR provided strong evidence of capping and crystallinity, future studies utilizing XPS could further elucidate the precise chemical states of the surface-bound biomolecules.

## Conclusion

According to the aforementioned data, it can be deduced that *R. sativus* root water extract consists of various bioactive constituents that are responsible for the conversion of gold ions into gold atoms and stabilizing it forming AuNPs in a green manner. The surface plasmon resonance peak of the ruby-red nanogold solution was at 526 nm. The spherical uneven shape of the formed gold nanoparticles and its distribution were determined by using AFM and TEM analysis. Size and zeta potential value of gold nanoparticles capped with *R. sativus* root extract were 31 nm with − 36.8mV, respectively. BET surface area of the formed mesoporous gold nanoparticles was 75.7187 m²/g and average pore size was 3.24442 nm. RRR-AuNPs exhibited stronger DPPH free radical scavenging activity than RRR extract, despite the latter having a higher total phenolic content. The RRR-AuNPs displayed an excellent antibacterial activity, inhibiting the growth of *S. aureus* and *E. coli* bacteria with lower MIC and MBC values than those recorded for the individual RRR extract. The in vitro anticancer potency of stabilized RRR-AuNPs was confirmed by the MTT assay against Caco-2 and HepG2 cell lines, achieving effective cytotoxicity at lower concentrations compared with the extract alone. Furthermore, the remarkably high antimicrobial selectivity index (SI) of the biosynthesized RRR-AuNPs underscores their superior biocompatibility and potentiality as highly selective antimicrobial agents with minimal host toxicity. Collectively, these findings demonstrate that *Raphanus sativus* can be used for the green synthesis of AuNPs leading to a more potent antioxidant, antimicrobial, and anticancer activities than the *Raphanus sativus* root aqueous extract itself. This may highlight the role of red radish root for green synthesis of AuNPs to obtain a compound considered as a rich source of bioactive phytochemicals with significant potentiality for promoting human health and therapeutic applications.

## Supplementary Information

Below is the link to the electronic supplementary material.

Supplementary Material 1

## Data Availability

Data supporting the findings of this study are provided in the Supplementary Information. Additional materials are available from the corresponding author upon reasonable request.
