# Supplementary figures and images for: Green synthesis of gold nanoparticles utilizing Raphanus sativus root extract: characterization and in vitro investigation of its antioxidant, antimicrobial and anticancer impact
Source: Sci Rep. 2026 Jul 3;16:20553. doi: 10.1038/s41598-026-60345-9 (PMC13332216; doi:10.1038/s41598-026-60345-9)

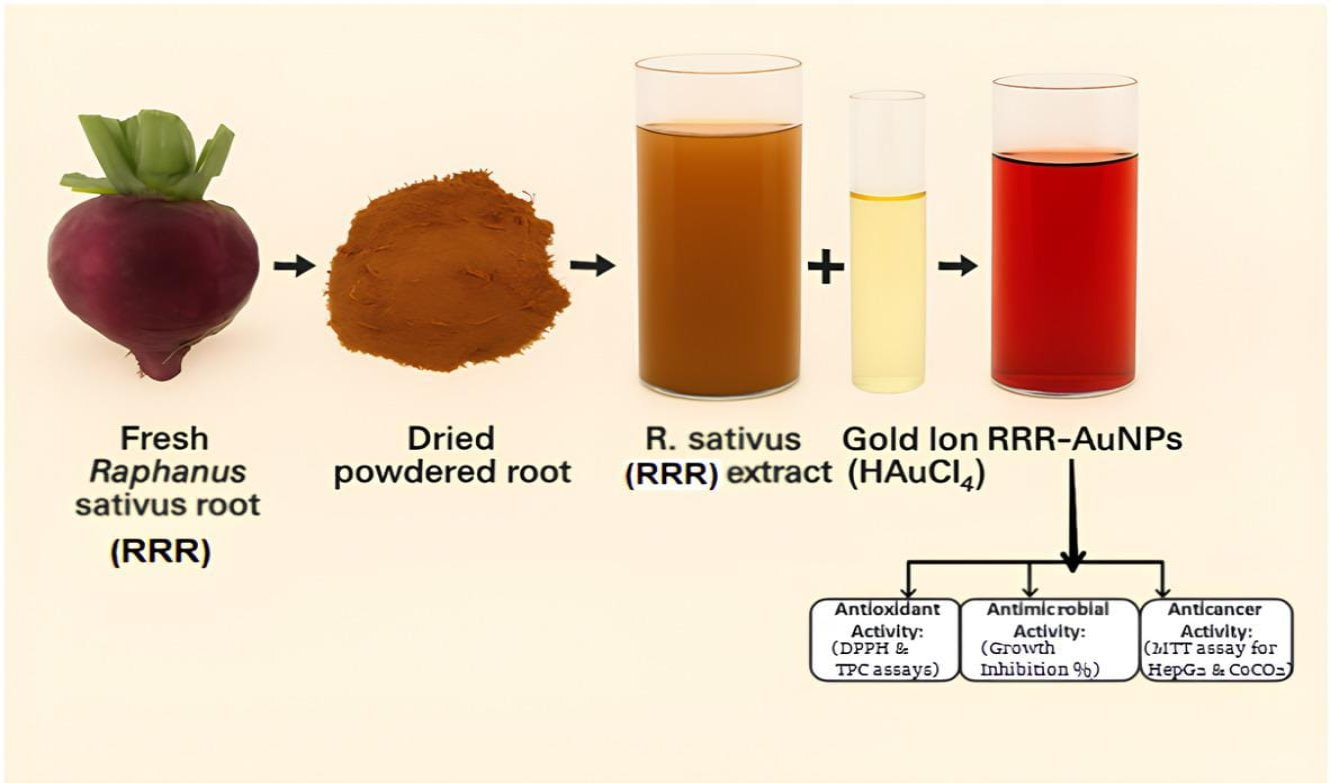

Supplement: Supplementary file 1 — Supplementary Material 1 [file 41598_2026_60345_MOESM1_ESM.tiff]
